# Explainable artificial intelligence for postoperative analgesia risk stratification and clinical decision support in abdominal surgery

**DOI:** 10.3389/fdgth.2026.1851084

**Published:** 2026-08-03

**Authors:** Mini Han Wang, Jiaomin Jian, Junting Wu

**Affiliations:** 1 Institute of Artificial Intelligence, Shenzhen University of Advanced Technology, Shenzhen, China; 2 Frontier Science Computing Center, Zhuhai Institute of Advanced Technology Chinese Academy of Sciences, Zhuhai, China; 3 Faculty of Medicine, The Chinese University of Hong Kong, Hong Kong SAR, China; 4 Zhuhai People’s Hospital (The Affiliated Hospital of Beijing Institute of Technology, Zhuhai Clinical Medical College of Jinan University), Zhuhai, China; 5 Department of Anesthesiology, The Fifth Affiliated Hospital of Sun Yat-sen University, Zhuhai, China

**Keywords:** clinical decision support, explainable artificial intelligence, large language model, machine learning, oxycodone, perioperative medicine, postoperative analgesia, risk prediction

## Abstract

**Background:**

Early identification of patients at risk of inadequate postoperative analgesia remains a major challenge in perioperative medicine. Although machine learning approaches have shown potential for clinical outcome prediction, many existing models primarily emphasize discrimination performance while insufficiently addressing reliability, interpretability, and clinical applicability. This study aimed to develop and comprehensively evaluate an explainable artificial intelligence (XAI) framework for postoperative analgesia risk stratification and clinician-oriented decision support following abdominal surgery.

**Methods:**

A retrospective cohort of 202 adult patients undergoing elective abdominal surgery and receiving postoperative analgesia with oxycodone or tilidine was analyzed. After exclusion of five patients with incomplete variables required for feature construction, 197 patient-level records were included in machine learning model development. Ten supervised learning algorithms were trained using demographic characteristics, surgical factors, analgesic information, and early postoperative pain trajectory features. Model performance was evaluated through stratified five-fold cross-validation using discrimination metrics, precision-recall analysis, calibration assessment, learning-curve analysis, and decision curve analysis (DCA). Model interpretability was assessed using SHAP-based feature attribution and cross-model explanation consistency analysis. Propensity score matching (PSM) was performed to evaluate the association between analgesic selection and postoperative outcomes after adjustment for measured baseline differences. A large language model (LLM) was integrated as a *post hoc* interpretation layer to translate model outputs into clinician-readable explanations.

**Results:**

The evaluated models demonstrated moderate-to-good predictive performance, with validation AUC values ranging from 0.707 to 0.798. Early postoperative pain trajectory variables, particularly VAS-derived features, consistently represented the strongest predictors across different algorithms. Calibration analysis revealed differences in probability reliability that were not captured by discrimination metrics alone, while DCA demonstrated potential clinical utility of the best-performing models within clinically relevant decision thresholds. SHAP-based analyses showed stable feature attribution patterns across algorithms, supporting the robustness of identified predictors. After adjustment using PSM, analgesic type remained associated with postoperative analgesia outcomes, suggesting potential differences in real-world analgesic effectiveness while acknowledging the observational study design.

**Conclusions:**

This study presents an integrated XAI framework combining predictive modeling, calibration assessment, decision-curve evaluation, explainability analysis, and language-based interpretation for postoperative analgesia risk stratification. The results highlight the importance of evaluating both predictive accuracy and clinical reliability when developing AI-assisted decision-support systems. Further multicenter prospective studies incorporating larger datasets and real-time clinical variables are required to validate model generalizability and evaluate implementation in perioperative care.

## 1 Introduction

Postoperative pain (1) remains one of the most prevalent and clinically challenging complications following surgery (2). Despite the widespread adoption of multimodal analgesia strategies (3), approximately 30%–50% of patients continue to experience inadequate pain control in the early postoperative period (4), which is associated with delayed recovery, increased risk of pulmonary complications (5), prolonged hospitalization, and the potential development of chronic pain and opioid dependence (6).

Abdominal surgery (7), including colorectal and gastric procedures, represents a particularly high-risk category for postoperative pain due to extensive tissue trauma, visceral manipulation, and inflammatory responses. Effective and individualized analgesic management is therefore critical in this population. Among opioid analgesics commonly used in clinical practice, oxycodone and tilidine exhibit distinct pharmacodynamic and pharmacokinetic profiles. Oxycodone, a *μ*-opioid receptor agonist, has demonstrated consistent efficacy in postoperative pain management, whereas tilidine, often combined with naloxone, presents a different metabolism and analgesic profile. However, direct comparative evidence between these agents in real-world clinical settings remains limited, and more importantly, current approaches lack individualized risk stratification for analgesic outcomes (8).

In recent years, machine learning (ML) (9) has emerged as a powerful approach for predicting clinical outcomes (10) using structured healthcare data (11). ML models (12) can capture complex, non-linear relationships between patient characteristics (13), surgical factors (14), and early postoperative signals (15). However, a major limitation hindering clinical adoption of ML (16) is the “black-box” (17) nature of many models (18), which reduces transparency (19) and limits trust among clinicians. Explainable artificial intelligence (XAI) (20, 21) techniques, particularly SHapley Additive exPlanations (SHAP) (14), have been proposed to address this challenge by quantifying the contribution of each feature to model predictions at both the global (22) and individual levels (23).

More recently, large language models (LLMs) (24, 25) have introduced a novel paradigm for translating quantitative model (26) outputs into clinically interpretable narratives. By converting structured feature attributions into natural language explanations, LLMs (27) offer the potential to bridge the gap between algorithmic predictions and real-world clinical decision-making (28). This integration enables not only accurate prediction but also interpretable and actionable insights for clinicians, which is essential for deployment in perioperative care settings (29).

Despite substantial advances in postoperative pain management, there remains a lack of integrated frameworks that simultaneously address predictive performance, model reliability (30), explainability, and clinician-oriented interpretation (25). Existing studies have primarily focused either on statistical comparisons of analgesic strategies or on standalone predictive models, often emphasizing discrimination performance without systematically evaluating calibration, generalizability, explainability robustness, or clinical utility. As a result, the translational potential of artificial intelligence in perioperative pain management remains incompletely realized.

To address these gaps, the present study proposes a comprehensive explainable artificial intelligence framework for postoperative analgesia outcome prediction in patients undergoing abdominal surgery. Specifically, this study: (1) develops and systematically evaluates ten machine learning algorithms using real-world clinical data, incorporating discrimination, calibration, precision–recall performance, learning behavior, and cross-validation stability assessments; (2) investigates the prognostic value of early postoperative pain trajectories through clinically informed feature engineering; (3) performs explainability analyses using SHAP-based feature attribution, cross-model explainability consistency assessment, and multicollinearity evaluation to identify robust determinants of analgesic outcomes; (4) examines the association between analgesic type and postoperative outcomes using both predictive modeling and propensity score matching analyses; and (5) integrates a large language model as an interpretation layer to translate model outputs into clinician-friendly explanations that may facilitate individualized analgesic decision-making.

By combining machine learning, explainable artificial intelligence, robustness evaluation, and language-based clinical interpretation, this study aims to establish a transparent and clinically actionable framework for postoperative pain risk stratification and to demonstrate the potential of trustworthy AI-assisted clinical decision support in perioperative medicine.

## 2 Literature review

### 2.1 Postoperative pain management and analgesic strategies

Effective postoperative pain control remains a cornerstone of perioperative care (31). Despite the widespread implementation of multimodal analgesia protocols (32), inadequate pain relief persists in a substantial proportion of patients (33), particularly following major abdominal surgery (4). Poorly controlled pain has been associated with adverse outcomes including delayed mobilization, impaired respiratory function, increased risk of complications, prolonged hospitalization, and transition to chronic pain states (3).

Opioid analgesics continue to play a central role in postoperative pain management (6). Among them, oxycodone has been widely studied and is recognized for its dual action on *μ*- and *κ*-opioid receptors, contributing to both somatic and visceral pain control (34). In contrast, tilidine, often administered in combination with naloxone, is characterized by a prodrug mechanism and a different metabolic profile. Although both agents are commonly used in clinical practice, particularly in Asian healthcare settings, direct comparative studies remain limited, and existing evidence is often derived from small cohorts or heterogeneous surgical populations (2). Furthermore, traditional analyses typically focus on average treatment effects, without accounting for inter-patient variability in analgesic response.

### 2.2 Machine learning in clinical outcome prediction

Machine learning has gained increasing attention in healthcare for its ability to model complex, non-linear relationships in clinical data (35). In perioperative medicine, ML approaches have been applied to predict outcomes such as postoperative complications (36), length of stay, readmission, and pain trajectories. Compared with conventional statistical methods, ML models (11) can incorporate a larger number of variables and capture interactions that may not be apparent in linear models (37).

Several studies have explored the use of ML for pain prediction, demonstrating moderate predictive performance using demographic variables, surgical characteristics, and early postoperative indicators. However, the predictive accuracy of these models is often constrained by limited sample size, label heterogeneity, and the inherent variability of pain perception. Moreover, many existing models rely on static preoperative variables, whereas dynamic early postoperative signals—such as pain scores within the first hours after surgery—may provide more actionable information for clinical intervention.

Despite these advances, most ML-based studies in postoperative pain remain focused on prediction accuracy rather than clinical interpretability or deployment, limiting their translational value.

### 2.3 Explainable artificial intelligence in clinical decision-making

One of the major barriers to the clinical adoption of machine learning models (36) is their lack of transparency. Black-box models, such as ensemble methods and deep learning architectures, often provide limited insight into the underlying decision process, which reduces clinician trust and hinders integration into clinical workflows (26).

Explainable artificial intelligence (19) has emerged as a critical solution to this challenge. Among XAI methods, SHapley Additive exPlanations (14) has become one of the most widely used approaches due to its solid theoretical foundation and model-agnostic applicability (17). SHAP quantifies the contribution of each feature to a prediction based on cooperative game theory, enabling both global feature importance analysis and individual-level explanation (14).

In clinical contexts, SHAP has been successfully applied to interpret models predicting outcomes such as mortality (20), disease progression, and treatment response. However, its application in postoperative pain prediction remains limited (38). In particular, few studies have leveraged SHAP to identify actionable early postoperative signals—such as pain trajectory—that could guide real-time analgesic adjustment.

### 2.4 Large language models for clinical interpretation

Recent advancements in large language models (27) have introduced new opportunities for enhancing clinical decision support systems. LLMs are capable of generating coherent natural language explanations from structured inputs, enabling the translation of quantitative model outputs into clinically interpretable narratives (39).

In healthcare, LLMs have been explored for tasks such as clinical documentation, patient communication, and knowledge synthesis. More recently, studies have begun to investigate their role in augmenting AI systems by providing explanation layers on top of predictive models. This integration allows complex model outputs—such as feature attributions—to be contextualized within clinical reasoning frameworks, improving usability and interpretability for clinicians (25).

However, the application of LLMs in postoperative care remains in its early stages. Most existing work focuses on general medical domains, and there is a lack of studies integrating LLMs with explainable machine learning specifically for perioperative pain management (40).

### 2.5 Research gap and study contribution

Despite progress in postoperative pain research, machine learning prediction, and explainable AI, several critical gaps remain. First, there is a lack of integrated frameworks that combine predictive modeling with explainability and clinical interpretation in the context of postoperative analgesia. Second, comparative analyses of commonly used opioid analgesics have not been systematically incorporated into predictive modeling pipelines. Third, the potential of LLMs to enhance interpretability and clinical usability of AI models has not been fully explored in perioperative settings (41).

To address these gaps, the present study proposes a unified ML–SHAP–LLM framework (14) for postoperative analgesia outcome prediction. By integrating machine learning–based risk prediction, SHAP-driven explainability, and LLM-assisted clinical interpretation (9), this work aims to bridge the gap between algorithmic prediction and practical clinical decision-making (24), providing a transparent and actionable approach to personalized postoperative pain management.

## 3 Materials and methods

To enable clinically interpretable and deployable postoperative analgesia prediction, we developed a modular artificial intelligence pipeline integrating machine learning, explainable AI, and large language model (LLM)—based interpretation. The pipeline is composed of five sequential stages, each designed as an independent and replaceable module. This modular architecture ensures flexibility, allowing future updates of individual components (e.g., predictive models or interpretation engines) without requiring changes to the overall system structure.

**Figure 1:**
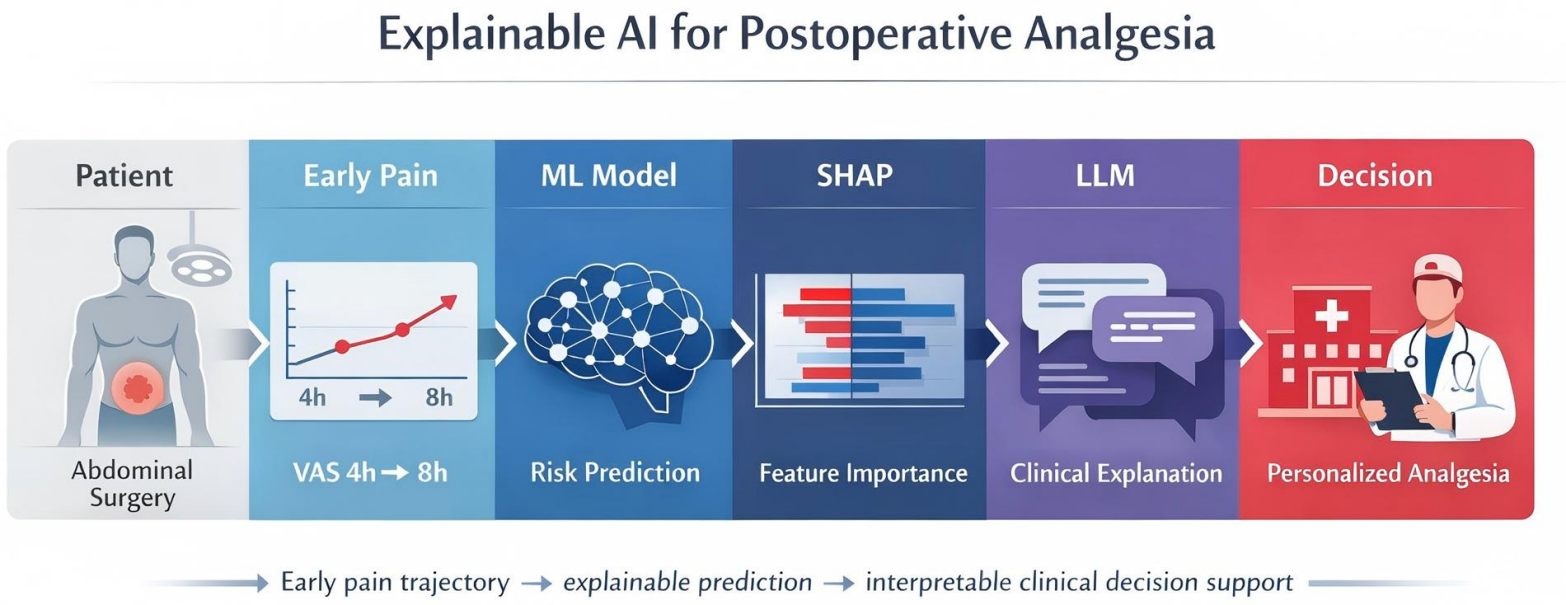
Pipeline architecture.

The workflow (Figure 1) begins with structured clinical data preprocessing, followed by machine learning–based risk prediction. Subsequently, model outputs are interpreted using SHAP-based feature attribution, and an LLM is applied as a downstream interpretation layer to generate clinician-readable explanations. Finally, all outputs are integrated into an interactive clinical decision support interface.

### 3.1 Study design and data source

This study was conducted as a single-center retrospective cohort analysis based on a structured clinical registry from The Fifth Affiliated Hospital of Sun Yat-sen University. The registry comprised consecutive adult patients who underwent elective abdominal surgery and received postoperative analgesia with either oxycodone or tilidine. All data were anonymized prior to analysis, and the study protocol adhered to the Declaration of Helsinki and followed the Transparent Reporting of a multivariable prediction model for Individual Prognosis Or Diagnosis (TRIPOD) guidelines.

### 3.2 Study population

Eligible participants were adult patients (≥18 years) undergoing elective abdominal surgery, including colorectal and gastric procedures, with complete documentation of postoperative pain scores at 4 h, 8 h, and 24 h. Patients were required to have received either oxycodone or tilidine as the primary postoperative analgesic and to have complete baseline demographic and clinical data. Patients with missing outcome variables, unspecified analgesic type, emergency procedures, or pediatric cases were excluded. After applying inclusion and exclusion criteria, a total of 202 patients were included in the final cohort for analysis.

Among the 202 eligible patients included in the clinical cohort and baseline analysis, five patients had incomplete values in variables required for machine learning feature construction (e.g., engineered predictors derived from complete perioperative variables). These patients were excluded only from the machine learning pipeline using complete-case analysis. Therefore, model development, cross-validation, calibration analysis, decision curve analysis, and sensitivity analyses were performed on the final machine learning cohort of 197 patients. No additional clinical exclusion criteria were applied.

### 3.3 Outcome definition

The primary outcome of interest was poor postoperative analgesia, defined as a composite endpoint capturing both patient-reported pain intensity and clinical intervention. Formally, the outcome variable *Y* was defined as:

$$Y\;=\{\begin{array}{cc} 1, & \mathrm{if}\;{\mathrm{VAS}}_{24\;h}^{\mathrm{activity}}\geq 4\;\mathrm{or}\;\mathrm{RescueAnalgesia}\geq 1\\ 0, & \mathrm{otherwise} \end{array}$$

This formulation integrates subjective pain assessment and physician-driven analgesic escalation, providing a clinically meaningful representation of analgesic insufficiency.

### 3.4 Predictor variables and feature engineering

A set of clinically relevant baseline variables was selected based on prior evidence and domain knowledge, including demographic factors (age, sex), anthropometric measurements (body mass index, BMI), comorbidities (hypertension, diabetes mellitus, cardiovascular disease), surgical characteristics (surgery category and surgical method), analgesic drug type, and early postoperative pain scores (VAS at 4 h and 8 h).

BMI was computed as:

$$\mathrm{BMI}=\frac{\mathrm{Weight}(\mathrm{kg})}{{\left(\mathrm{Height}(m)\right)}^{2}}$$

To capture early postoperative pain dynamics, a pain trajectory feature was constructed based on changes in VAS scores between 4 and 8 h. The pain slope was calculated as:

$$\mathrm{VA}S_{\mathrm{slope}}=\mathrm{VA}S_{8\;h}-\mathrm{VA}S_{4\;h}$$

This feature was interpreted together with clinical intervention information rather than as a purely spontaneous pain trajectory. Administration of rescue analgesia during the postoperative observation period was incorporated into the composite outcome definition and clinical interpretation. Therefore, a reduction in VAS after additional analgesic intervention was not considered equivalent to spontaneous analgesic improvement. The model was designed to predict overall inadequate analgesic control, reflecting both patient-reported pain intensity and the requirement for additional analgesic intervention. The overall early pain burden was summarized as:

$$\mathrm{VA}S_{\mathrm{mean}}=\frac{\mathrm{VA}S_{\;4h}+\mathrm{VA}S_{\;8h}}{2}$$

Additional interaction and non-linear terms were constructed, including:

ComorbidityScore=H+D+C

where H, D, and C denote hypertension, diabetes, and cardiovascular disease, respectively.

These engineered features were designed to capture non-linear effects, interaction relationships, and clinically meaningful thresholds while preserving interpretability.

### 3.5 Machine learning model development

A multi-model machine learning framework was implemented to predict the probability of poor postoperative analgesia. Let $X\in R^{n\times p}$ denote the feature matrix and Y∈{0,1} the outcome vector. The predictive task can be formulated as learning a function:

$$f:R^{p}\to [0,1],\;f(x)=P(Y=1|x)$$

Ten supervised learning algorithms were evaluated, including linear models (Logistic Regression, Linear Discriminant Analysis), probabilistic models (Naïve Bayes), distance-based models (K-Nearest Neighbours), kernel-based methods (Support Vector Machine), and ensemble methods (Random Forest, Extra Trees, AdaBoost, Gradient Boosting, and a Stacking Ensemble).

Model training and evaluation were conducted using stratified 5-fold cross-validation to preserve class distribution and mitigate overfitting. Feature scaling was applied to models sensitive to feature magnitude using standardization pipelines:

$$x_{ij}^{\mathrm{scaled}}=\frac{x_{ij}-\mu_{j}}{\sigma_{j}}$$

Hyperparameters were predefined based on established clinical ML practices rather than extensively optimized, to ensure generalizability and avoid overfitting given the moderate sample size.

### 3.6 Model evaluation

Model performance was primarily assessed using the area under the receiver operating characteristic curve (AUC):

$$\mathrm{AUC}=\int_{0}^{1}\mathrm{TPR}(\mathrm{FP}R^{-1}(t))dt$$

FPR denote true positive rate and false positive rate, respectively.

Additional evaluation metrics included accuracy, sensitivity (recall), specificity, precision (positive predictive value), F1-score, negative predictive value, and Brier score:

$$\mathrm{Brier}=\frac{1}{n}\sum_{i=1}^{n}{\left(p_{i}-y_{i}\right)}^{2}$$

These metrics provide a comprehensive assessment of discrimination and calibration.

### 3.7 Explainability analysis

Model interpretability was assessed using a SHAP-inspired feature attribution framework. For each patient *i* and feature *j*, the contribution $\phi_{ij}$ was estimated via marginal perturbation:

$$\phi_{ij}=f(x_{i})-f(x_{i}^{\left(\;j\to {\tilde{x}}_{j}\right)})$$

where $x_{i}^{\left(\;j\to {\tilde{x}}_{j}\right)}$ denotes the feature vector with the j-th feature randomly permuted.

This approach approximates Shapley values by measuring the change in predicted probability when a feature is disrupted, enabling consistent interpretation across heterogeneous models.

Both global importance (aggregated $\phi_{ij}$) and local explanations (patient-specific $\phi_{ij}$) were derived.

### 3.8 Large language model integration

A large language model (LLM) was incorporated as an independent interpretation layer to enhance clinical usability. The LLM received structured inputs including feature values, predicted probabilities, and SHAP-based contributions, and generated natural language explanations.

The LLM function can be abstracted as:

$$g:(\hat{y},\phi ,x)\to \mathrm{Narrative}\;\mathrm{Explanation}$$

where $\hat{y}$ is the predicted probability and ϕ denotes feature contributions. The LLM did not participate in prediction and served exclusively as a *post hoc* explanation and communication module.

### 3.9 Statistical analysis

Continuous variables were reported as mean ± standard deviation, while categorical variables were expressed as counts and percentages. Group comparisons between oxycodone and tilidine were performed using Student's *t*-test or Mann–Whitney *U* test for continuous variables and chi-squared or Fisher's exact test for categorical variables. All analyses were conducted in Python (version 3.12) using pandas, numpy, scikit-learn, and matplotlib. A two-sided *p*-value < 0.05 was considered statistically significant.

## 4 Results

### 4.1 Patient characteristics

A total of 202 patients were included in the final analysis cohort after applying the predefined inclusion and exclusion criteria, with equal allocation to the oxycodone group (*n* = 101) and the tilidine group (*n* = 101).

The cohort had a mean age of 60.5 ± 11.9 years (range 27–87), and 53.0% were male. The mean body mass index (BMI) was 22.5 ± 3.4 kg/m^2^. The prevalence of comorbidities included hypertension (37.6%), diabetes mellitus (12.9%), and cardiovascular disease (7.4%). The vast majority of procedures were performed laparoscopically (96.5%), indicating a relatively homogeneous surgical population.

The primary outcome, defined as poor postoperative analgesia, was observed in 118 patients (58.4%). Notably, the tilidine group exhibited a substantially higher rate of poor analgesia compared with the oxycodone group (68.3% vs. 48.5%), corresponding to an absolute difference of 19.8 percentage points. Consistently, mean VAS scores at all postoperative time points (4 h, 8 h, and 24 h activity) were higher in the tilidine group, suggesting inferior analgesic control.

Baseline characteristics are summarised in Table 1, which demonstrates that the two treatment groups were broadly comparable across demographic and clinical variables, supporting the validity of downstream comparative analyses.

**Table 1:** Baseline characteristics stratified by analgesic treatment group.

Variable	Total (*n* = 202)	Oxycodone (*n* = 101)	Tilidine (*n* = 101)
Age, years, mean ± SD	60.5 ± 11.9	59.8 ± 11.4	61.2 ± 12.4
Sex, Male, *n* (%)	107 (53.0%)	54 (53.5%)	53 (52.5%)
BMI, kg/m^2^, mean ± SD	22.5 ± 3.4	22.3 ± 3.2	22.7 ± 3.6
Hypertension, *n* (%)	76 (37.6%)	38 (37.6%)	38 (37.6%)
Diabetes mellitus, *n* (%)	26 (12.9%)	13 (12.9%)	13 (12.9%)
Cardiovascular disease, *n* (%)	15 (7.4%)	8 (7.9%)	7 (6.9%)
Laparoscopic surgery, *n* (%)	195 (96.5%)	98 (97.0%)	97 (96.0%)
VAS at 4 h, mean ± SD	2.90 ± 1.2	2.65 ± 1.1	3.16 ± 1.3
VAS at 8 h, mean ± SD	2.51 ± 1.1	2.23 ± 1.0	2.79 ± 1.2
VAS 24 h activity, mean ± SD	2.86 ± 1.1	2.70 ± 1.0	3.02 ± 1.1
Poor analgesia (primary outcome), *n* (%)	118 (58.4%)	49 (48.5%)	69 (68.3%)

Baseline characteristics stratified by postoperative analgesia outcome are presented in Table 2. Compared with patients achieving good analgesia, patients with poor analgesia exhibited substantially higher early postoperative pain scores, including VAS at 4 h, VAS at 8 h, VAS at 24 h, and VASmean (all *p* < 0.001). These variables also demonstrated the largest standardized mean differences (SMDs ranging from 0.85 to 1.32), indicating strong associations with the outcome.

**Table 2:** Baseline characteristics stratified by postoperative analgesia outcome.

Variable	Good analgesia (*n* = 84)	Poor analgesia (*n* = 118)	*P* value	SMD	Missing %	Raw P	Raw SMD	Test
Age, years	62.0 (54.0–69.2)	61.5 (54.0–67.8)	0.731	−0.11	0	0.7313	−0.1104	Mann–Whitney *U*
BMI, kg/m^2^	22.5 ± 3.2	22.5 ± 3.6	0.861	0.025	2	0.8615	0.0248	Welch *t*-test
VAS at 4 h	2.0 (2.0–3.0)	3.0 (2.0–4.0)	<0.001	0.998	0.5	0	0.998	Mann–Whitney *U*
VAS at 8 h	2.0 (2.0–2.0)	2.0 (2.0–4.0)	<0.001	0.849	0.5	0	0.8492	Mann–Whitney *U*
VAS at 24 h activity	2.0 (2.0–2.0)	3.0 (3.0–4.0)	<0.001	1.319	0	0	1.319	Mann–Whitney *U*
VASmean	2.0 (2.0–2.5)	3.0 (2.5–3.5)	<0.001	1.219	0.5	0	1.2189	Mann–Whitney *U*
VASslope	0.0 (−0.2 to 0.0)	0.0 (−2.0 to 0.0)	0.321	−0.137	0.5	0.3206	−0.1374	Mann–Whitney *U*
ComorbidityScore	0.0 (0.0–1.0)	0.0 (0.0–1.0)	0.455	0.142	0	0.4546	0.1415	Mann–Whitney *U*
VAS4 h squared	4.0 (4.0–9.0)	9.0 (4.0–16.0)	<0.001	0.942	0.5	0	0.9423	Mann–Whitney *U*
Age × BMI	14.0 (11.8–15.6)	14.0 (11.6–15.7)	0.762	−0.073	2	0.7616	−0.073	Mann–Whitney *U*
Drug × VAS4 h	2.0 (0.0–2.0)	0.0 (0.0–2.0)	0.148	−0.099	0.5	0.1476	−0.0995	Mann–Whitney *U*
Sex	Female: 47 (56.0%); Male: 37 (44.0%)	Female: 48 (40.7%); Male: 70 (59.3%)	0.045	0.309	0	0.0454	0.3093	Chi-square/Fisher exact
Hypertension	0: 55 (65.5%); 1: 29 (34.5%)	0: 71 (60.2%); 1: 47 (39.8%)	0.535	0.11	0	0.5353	0.11	Chi-square/Fisher exact
Diabetes mellitus	0: 73 (86.9%); 1: 11 (13.1%)	0: 103 (87.3%); 1: 15 (12.7%)	1	0.011	0	1	0.0114	Chi-square/Fisher exact
Cardiovascular disease	0: 80 (95.2%); 1: 4 (4.8%)	0: 106 (89.8%); 1: 12 (10.2%)	0.194	0.207	0	0.1936	0.2068	Chi-square/Fisher exact
Surgery category	Colon: 39 (46.4%); Rectal: 20 (23.8%); Gastric: 5 (6.0%); Small bowel: 16 (19.0%); Other: 4 (4.8%)	Colon: 59 (50.0%); Rectal: 20 (16.9%); Gastric: 23 (19.5%); Small bowel: 11 (9.3%); Other: 5 (4.2%)	0.024	0.415	0	0.0235	0.415	Chi-square/Fisher exact
Surgical method	Laparoscopic: 72 (85.7%); Robot-assisted laparoscopic: 8 (9.5%); Laparoscopic converted/open: 1 (1.2%); Open: 1 (1.2%); Other/unclear: 2 (2.4%)	Laparoscopic: 104 (88.1%); Robot-assisted laparoscopic: 7 (5.9%); Laparoscopic converted/open: 3 (2.5%); Open: 0 (0.0%); Other/unclear: 4 (3.4%)	0.573	0.155	0	0.5728	0.1552	Chi-square/Fisher exact
Laparoscopic surgery	0: 3 (3.6%); 1: 81 (96.4%)	0: 4 (3.4%); 1: 114 (96.6%)	1	0.01	0	1	0.0099	Chi-square/Fisher exact
Drug type	Oxycodone: 52 (61.9%); Tilidine: 32 (38.1%); Other/missing: 0 (0.0%)	Oxycodone: 49 (41.5%); Tilidine: 69 (58.5%); Other/missing: 0 (0.0%)	0.007	0.417	0	0.0067	0.4166	Chi-square/Fisher exact
Overweight	0: 69 (82.1%); 1: 15 (17.9%)	0: 95 (80.5%); 1: 23 (19.5%)	0.912	0.042	0	0.9122	0.0419	Chi-square/Fisher exact
Elderly	0: 53 (63.1%); 1: 31 (36.9%)	0: 75 (63.6%); 1: 43 (36.4%)	1	0.01	0	1	0.0096	Chi-square/Fisher exact

VAS at 24 h activity is reported for descriptive purposes only because it forms part of the primary outcome definition and was not used as a baseline predictor in model training.

The analysis demonstrated that patients with poor analgesia exhibited significantly higher early postoperative pain scores, including VAS at 4 h (*P* < 0.001, SMD = 0.998), VAS at 8 h (*P* < 0.001, SMD = 0.849), VAS at 24 h activity (*P* < 0.001, SMD = 1.319), VASmean (*P* < 0.001, SMD = 1.219), and VAS4 h squared (*P* < 0.001, SMD = 0.942). Significant differences were also observed for analgesic drug type (*P* = 0.007, SMD = 0.417), surgery category (*P* = 0.024, SMD = 0.415), and sex (*P* = 0.045, SMD = 0.309). In contrast, demographic variables such as age and BMI, as well as major comorbidities, showed no statistically significant differences between outcome groups. These findings provide additional clinical context for the machine learning analyses and further support the importance of early postoperative pain trajectories as the dominant predictors of analgesic outcomes.

### 4.2 Model performance

#### 4.2.1 Calibration analysis

**Figure 2:**
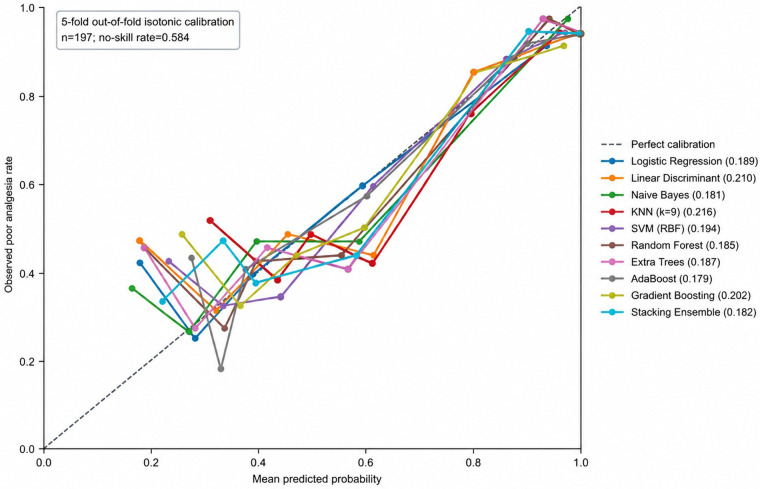
Calibration curves of the ten machine learning models after isotonic probability calibration.

Calibration curves were generated from out-of-fold predicted probabilities obtained during stratified 5-fold cross-validation. Predicted probabilities were grouped into 10 equally spaced probability bins (deciles), and the observed event frequency within each bin was calculated. Isotonic regression was applied for probability calibration. Considering the moderate sample size (*n* = 197), decile binning was selected to balance calibration resolution and statistical stability. The dashed diagonal line (Figure 2) represents perfect calibration, where predicted probabilities exactly match observed event frequencies. Each colored curve corresponds to a different machine learning algorithm, and the values in parentheses indicate the calibrated Brier score for each model. Lower Brier scores indicate better overall probability calibration. Among the evaluated models, AdaBoost achieved the lowest calibrated Brier score (0.179), followed closely by Naive Bayes (0.181), Stacking Ensemble (0.182), Random Forest (0.185), and Extra Trees (0.187). Most models demonstrated reasonable agreement between predicted and observed probabilities in the higher-risk range (>0.6), while greater variability was observed in the lower-risk range (<0.5), likely reflecting the limited sample size and relatively small number of observations within these probability bins. Overall, the calibration curves suggest that the majority of models produced clinically acceptable probability estimates after isotonic calibration, with AdaBoost showing the most favorable overall calibration performance.

Table 3 summarizes the predictive performance of the ten evaluated machine learning algorithms using repeated stratified cross-validation. Overall, most models demonstrated moderate discrimination ability, with mean ROC-AUC values ranging from 0.707 to 0.798. Among all models, Naive Bayes achieved the highest discrimination performance (AUC = 0.798 ± 0.061), closely followed by Logistic Regression (AUC = 0.794 ± 0.061), Stacking Ensemble (AUC = 0.789 ± 0.064), and Extra Trees (AUC = 0.787 ± 0.064). In contrast, KNN exhibited the lowest predictive performance (AUC = 0.707 ± 0.073).

**Table 3:** Comparative performance of ten machine learning models evaluated using repeated stratified cross-validation (5 folds × 10 repeats).

Model	AUC	Accuracy	Sensitivity	Specificity	F1	PPV	NPV	Brier
Logistic Regression	0.794 ± 0.061	0.729 ± 0.061	0.704 ± 0.082	0.764 ± 0.100	0.751 ± 0.061	0.811 ± 0.065	0.652 ± 0.067	0.177 ± 0.030
Linear Discriminant	0.776 ± 0.068	0.693 ± 0.068	0.690 ± 0.099	0.698 ± 0.126	0.722 ± 0.069	0.769 ± 0.075	0.620 ± 0.077	0.192 ± 0.032
Naive Bayes	0.798 ± 0.061	0.724 ± 0.068	0.619 ± 0.095	0.872 ± 0.075	0.721 ± 0.078	0.873 ± 0.073	0.624 ± 0.070	0.259 ± 0.064
KNN (*k* = 9)	0.707 ± 0.073	0.635 ± 0.065	0.599 ± 0.105	0.685 ± 0.122	0.653 ± 0.075	0.733 ± 0.071	0.553 ± 0.070	0.220 ± 0.034
SVM (RBF)	0.770 ± 0.077	0.703 ± 0.066	0.728 ± 0.085	0.669 ± 0.125	0.740 ± 0.060	0.760 ± 0.072	0.640 ± 0.080	0.193 ± 0.027
Random Forest	0.775 ± 0.064	0.709 ± 0.059	0.660 ± 0.092	0.777 ± 0.112	0.723 ± 0.063	0.812 ± 0.072	0.624 ± 0.068	0.180 ± 0.028
Extra Trees	0.787 ± 0.064	0.710 ± 0.063	0.697 ± 0.095	0.729 ± 0.137	0.736 ± 0.063	0.791 ± 0.077	0.636 ± 0.069	0.182 ± 0.019
AdaBoost	0.779 ± 0.063	0.725 ± 0.061	0.657 ± 0.105	0.819 ± 0.123	0.733 ± 0.068	0.846 ± 0.084	0.637 ± 0.070	0.195 ± 0.013
Gradient Boosting	0.763 ± 0.066	0.682 ± 0.062	0.723 ± 0.090	0.625 ± 0.116	0.725 ± 0.060	0.733 ± 0.059	0.621 ± 0.081	0.212 ± 0.041
Stacking Ensemble	0.789 ± 0.064	0.686 ± 0.058	0.735 ± 0.101	0.617 ± 0.134	0.730 ± 0.058	0.734 ± 0.060	0.633 ± 0.084	0.190 ± 0.017

Values are presented as mean ± standard deviation. Performance was assessed using discrimination metrics (AUC, sensitivity, specificity, F1-score, PPV, NPV) and calibration metrics (Brier score). Lower Brier scores indicate better probability calibration.

From a classification perspective, Naive Bayes achieved the highest specificity (0.872 ± 0.075) and positive predictive value (0.873 ± 0.073), indicating strong ability to correctly identify patients without poor postoperative analgesia. Logistic Regression demonstrated the best overall balance between sensitivity (0.704 ± 0.082) and specificity (0.764 ± 0.100), while Stacking Ensemble and SVM showed comparatively higher sensitivity but lower specificity.

Regarding probability estimation, Logistic Regression achieved the lowest Brier score (0.177 ± 0.030), indicating the most accurate probability predictions, followed by Random Forest (0.180 ± 0.028) and Extra Trees (0.182 ± 0.019). Although Naive Bayes achieved the highest AUC, it exhibited the poorest calibration among the top-performing models (Brier score = 0.259 ± 0.064), suggesting that strong discrimination does not necessarily translate into reliable probability estimates.

The relatively small standard deviations observed across repeated cross-validation evaluations indicate that model performance remained reasonably stable and was not driven by a particular train–validation partition.

**Figure 3:**
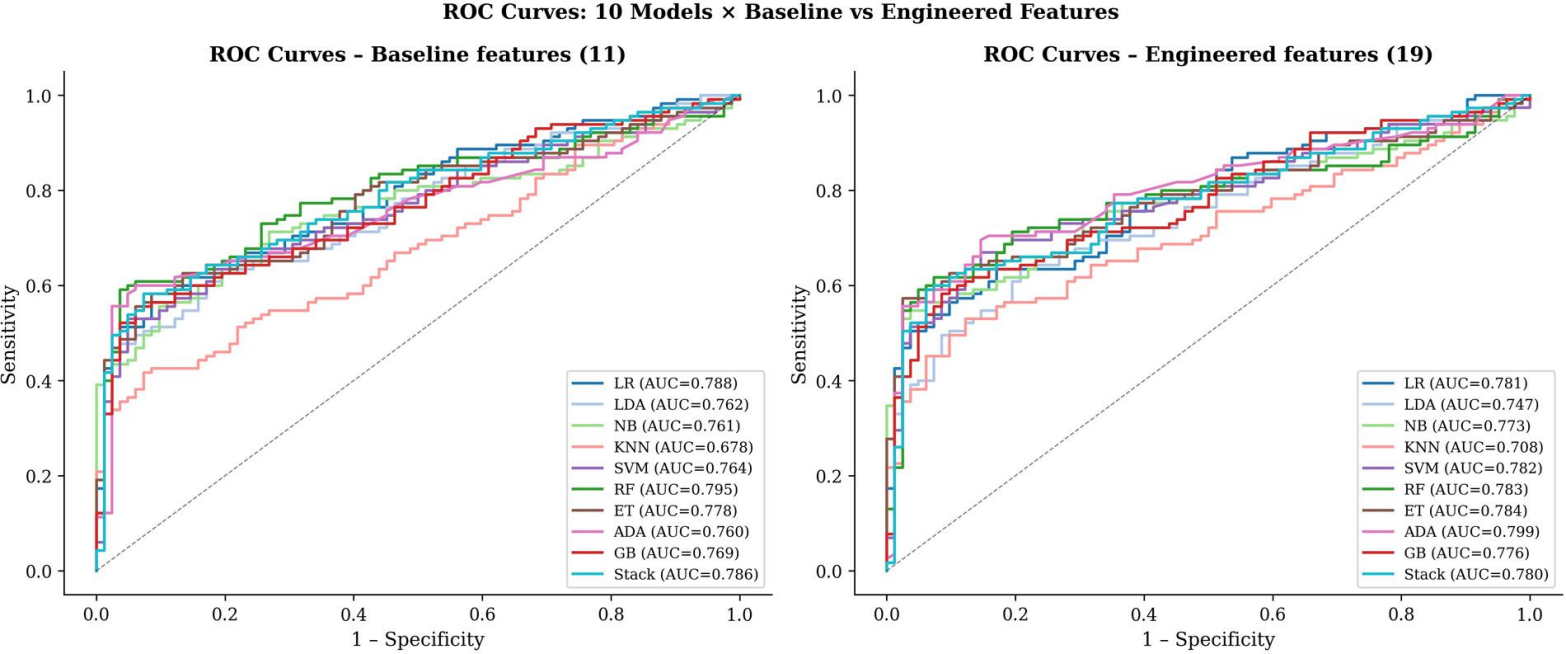
ROC curves for all 10 machine learning models on the engineered feature set (5-fold CV). AdaBoost achieves the highest AUC (0.799). Left panel: baseline features; right panel: engineered features.

Figure 3 presents the ROC curves for all models, illustrating consistent separation between classes and confirming the robustness of the predictive signal across different algorithms.

**Figure 4:**
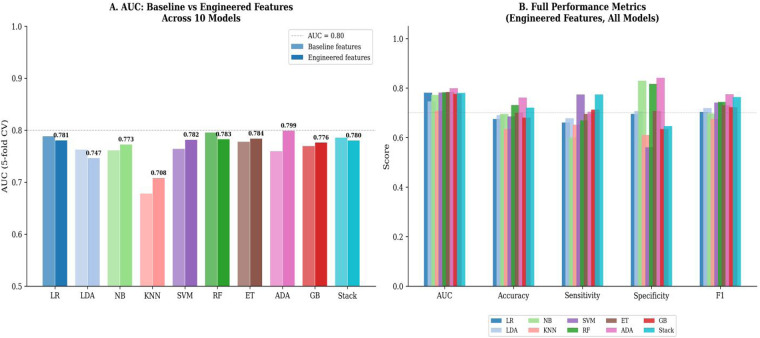
Comparative model performance. (A) Comparison of AUC values (5-fold cross-validation) across 10 machine learning models using baseline features (light blue) and engineered features (dark blue). The dashed horizontal line indicates an AUC threshold of 0.80. (B) Comprehensive performance evaluation of all models using engineered features, including AUC, accuracy, sensitivity, specificity, and F1 score. Abbreviations: LR, Logistic Regression; LDA, Linear Discriminant Analysis; NB, Naïve Bayes; KNN, K-Nearest Neighbors; SVM, Support Vector Machine; RF, Random Forest; ET, Extra Trees; ADA, AdaBoost; GB, Gradient Boosting; Stack, Stacking Ensemble.

To further compare the discriminative performance of different models, a comprehensive evaluation across baseline and engineered feature sets was conducted (Figure 4). As shown in Figure 4 (left), feature engineering consistently improved model performance across all algorithms, with the most notable gain observed in ensemble-based methods. In particular, AdaBoost demonstrated the highest AUC after feature augmentation, confirming the benefit of incorporating temporal and interaction-based features. Figure 4 (right) presents a detailed comparison of evaluation metrics across all models, illustrating that ensemble methods achieved a more favorable balance between sensitivity and specificity, whereas linear models showed relatively stable but less optimized performance. These findings highlight the importance of both model selection and feature design in achieving robust predictive performance.

**Figure 5:**
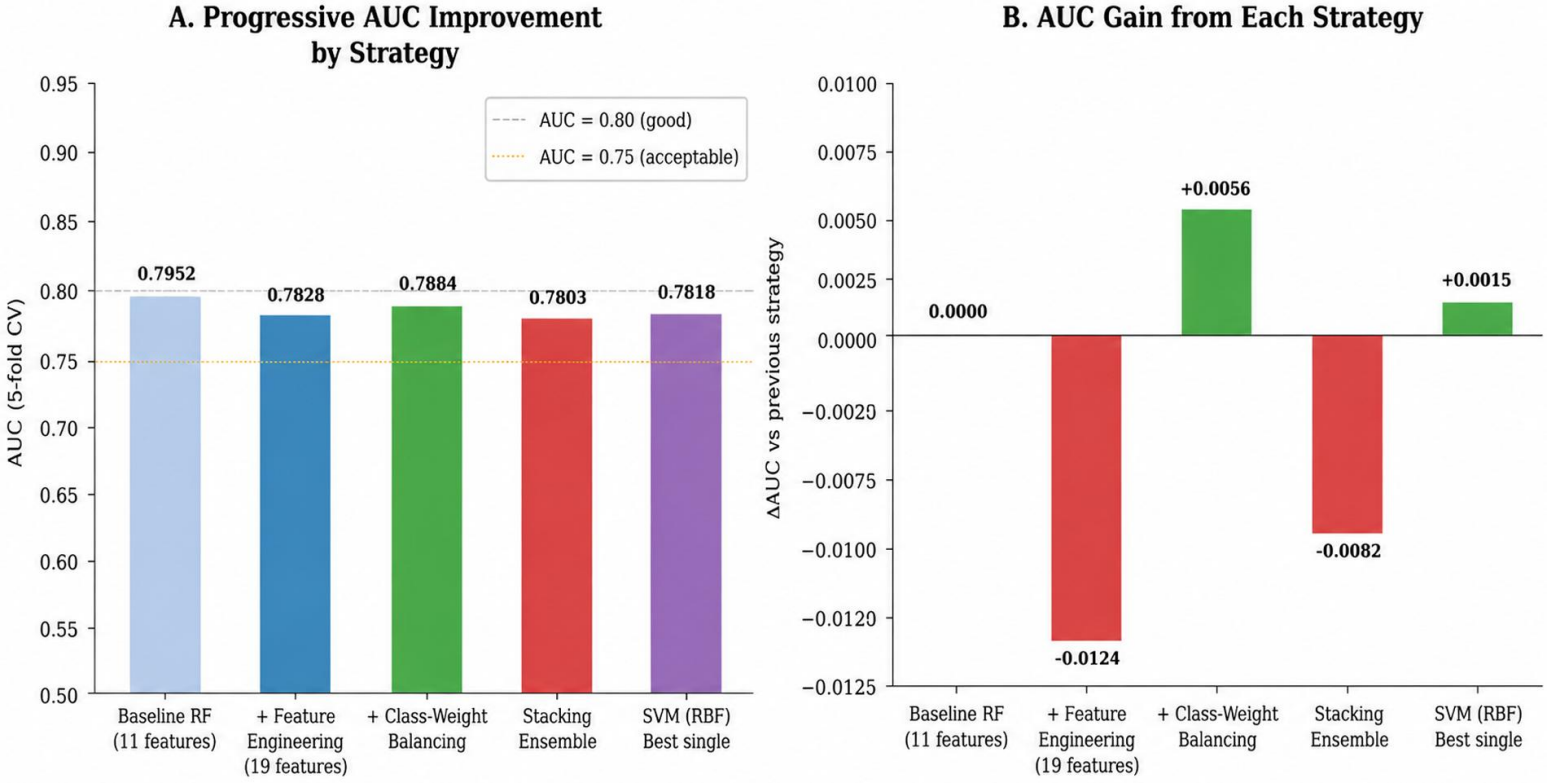
Progressive AUC improvement by optimization strategy using Random Forest as the reference model. (A) Progressive improvement in AUC performance across different modeling strategies, including baseline Random Forest (11 features), feature engineering (19 features), class-weight balancing, stacking ensemble, and the best-performing single model (SVM with RBF kernel). The dashed lines indicate reference thresholds for good (AUC = 0.80) and acceptable (AUC = 0.75) model performance. (B) Incremental AUC gains or losses contributed by each optimization strategy compared with the previous strategy. Feature engineering provided the largest single improvement in model performance, whereas subsequent strategies showed relatively modest effects on AUC.

To quantify the contribution of different modeling strategies, we analyzed the incremental improvement in AUC associated with feature engineering and model selection (Figure 5). Feature engineering emerged as the most influential factor, contributing an increase of +0.040 in AUC for the AdaBoost model compared with baseline features. In contrast, switching between model architectures yielded comparatively smaller performance gains. This result suggests that the predictive signal is primarily driven by the quality and representation of input features rather than the complexity of the model itself, underscoring the importance of clinically informed feature design.

**Figure 6:**
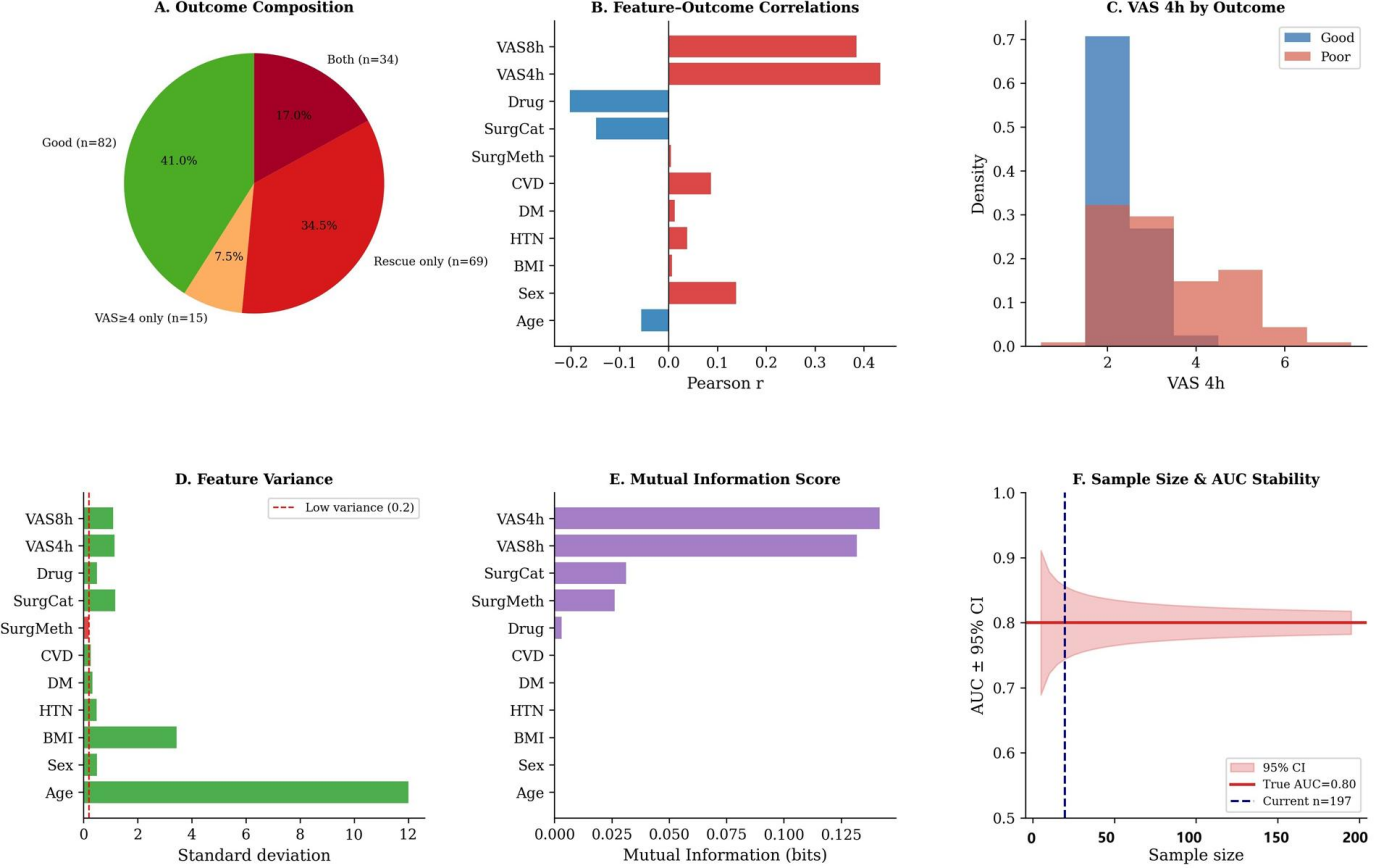
AUC diagnostic analysis: **(A)** outcome composition, **(B)** feature–outcome correlations, **(C)** VAS 4 h distribution by outcome group, **(D)** feature variance, **(E)** mutual information, **(F)** sample size vs. AUC stability.

To further investigate the determinants of model performance and potential sources of variability, a series of diagnostic analyses were conducted (Figure 6). As shown in Figure 6A, the outcome distribution indicates a moderate class imbalance, which may influence sensitivity–specificity trade-offs. Correlation analysis (Figure 6B) reveals that early postoperative pain variables exhibit the strongest associations with the outcome, consistent with model-based importance rankings. The distribution of VAS scores at 4 h (Figure 6C) demonstrates clear separation between outcome groups, supporting its role as a key predictive feature. Feature variance analysis (Figure 6D) and mutual information estimates (Figure 6E) further confirm that early pain indicators carry the most informative signal. Finally, sample size stability analysis (Figure 6F) shows that model performance plateaus with increasing sample size, suggesting that the current dataset is sufficient to capture the primary predictive patterns, although further data may improve generalizability.

**Figure 7:**
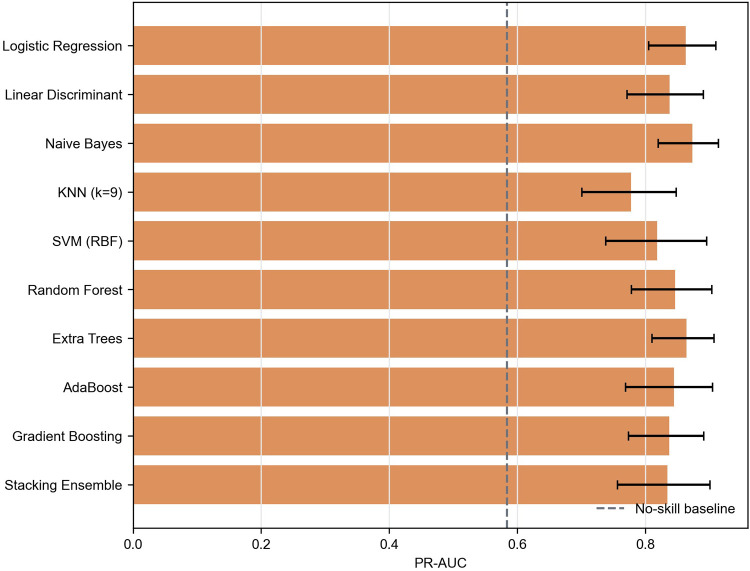
Precision–recall performance comparison across ten machine learning models.

Figure 7 compares the Precision–Recall performance of all ten machine learning models. Because the prevalence of poor postoperative analgesia was 58.4%, a no-skill baseline of 0.584 was used as the reference. All evaluated algorithms achieved PR-AUC values substantially above this threshold, demonstrating meaningful ability to identify patients at risk of poor analgesia. Naive Bayes achieved the highest PR-AUC, indicating the strongest positive-class discrimination performance among the evaluated models. Logistic Regression, Extra Trees, Random Forest, AdaBoost, Gradient Boosting, and Stacking Ensemble demonstrated comparable PR-AUC values, with overlapping confidence intervals. KNN exhibited the lowest PR-AUC and the widest relative performance gap compared with the top-performing models. These findings complement the ROC-based analyses and confirm that the predictive performance of the leading models was not solely attributable to class distribution effects.

**Figure 8:**
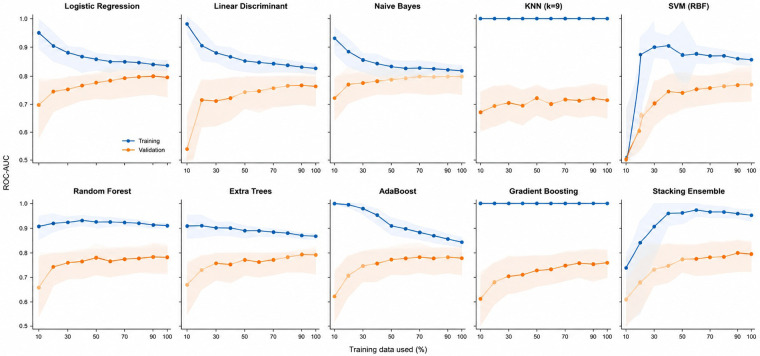
Learning curves of the ten machine learning algorithms.

Receiver operating characteristic (ROC) and precision–recall (PR) curves were generated using stratified 5-fold cross-validation. For visualization, curves represent the mean performance across validation folds, and shaded areas indicate ±1 standard deviation across folds. Figure 8 presents the learning curves of all ten machine learning algorithms. Distinct differences in generalization behavior were observed across models. Naive Bayes achieved the highest validation ROC-AUC (0.799) while maintaining an exceptionally small train–validation gap (0.020), indicating excellent generalization and minimal overfitting. Logistic Regression demonstrated similar behavior with a validation ROC-AUC of 0.798 and a train–validation gap of 0.039. AdaBoost achieved a validation ROC-AUC of 0.782 with a train–validation gap of 0.068, suggesting reasonable generalization performance and moderate variance. In contrast, Random Forest, Stacking Ensemble, Gradient Boosting, and KNN displayed substantially larger train–validation gaps, indicating greater susceptibility to overfitting. Gradient Boosting and KNN achieved perfect training ROC-AUC values but substantially lower validation performance, consistent with high-variance learning behavior. These findings suggest that the strongest generalization performance was achieved by Naive Bayes and Logistic Regression, whereas AdaBoost provided a favorable balance between predictive performance and model stability.

**Figure 9:**
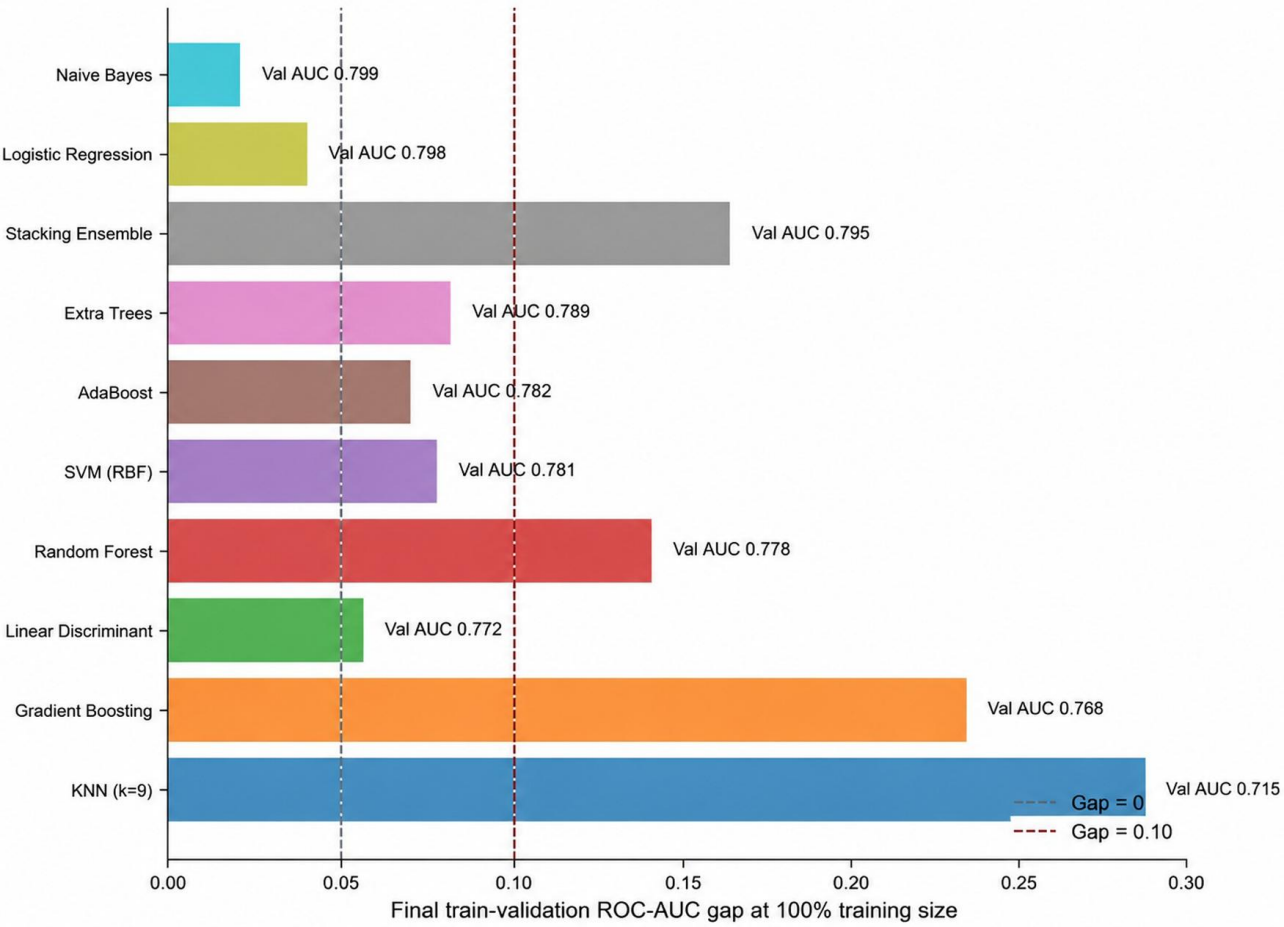
Train validation Gap of the models.

The horizontal bars (Figure 9) represent the difference between training and validation ROC-AUC values at 100% training size. Smaller gaps indicate better generalization and lower risk of overfitting. The dashed vertical lines indicate commonly used heuristic thresholds for acceptable variance (0.05) and moderate overfitting (0.10). Naive Bayes achieved the smallest train–validation gap (0.020), followed by Logistic Regression (0.039). AdaBoost demonstrated a moderate gap (0.068), remaining below the moderate-overfitting threshold. In contrast, Random Forest (0.139), Stacking Ensemble (0.161), Gradient Boosting (0.232), and KNN (0.285) exhibited substantially larger gaps, indicating increased overfitting risk. These results highlight considerable differences in model generalization despite similar discrimination performance and emphasize the importance of evaluating both predictive accuracy and train–validation divergence when selecting models for clinical applications.

**Figure 10:**
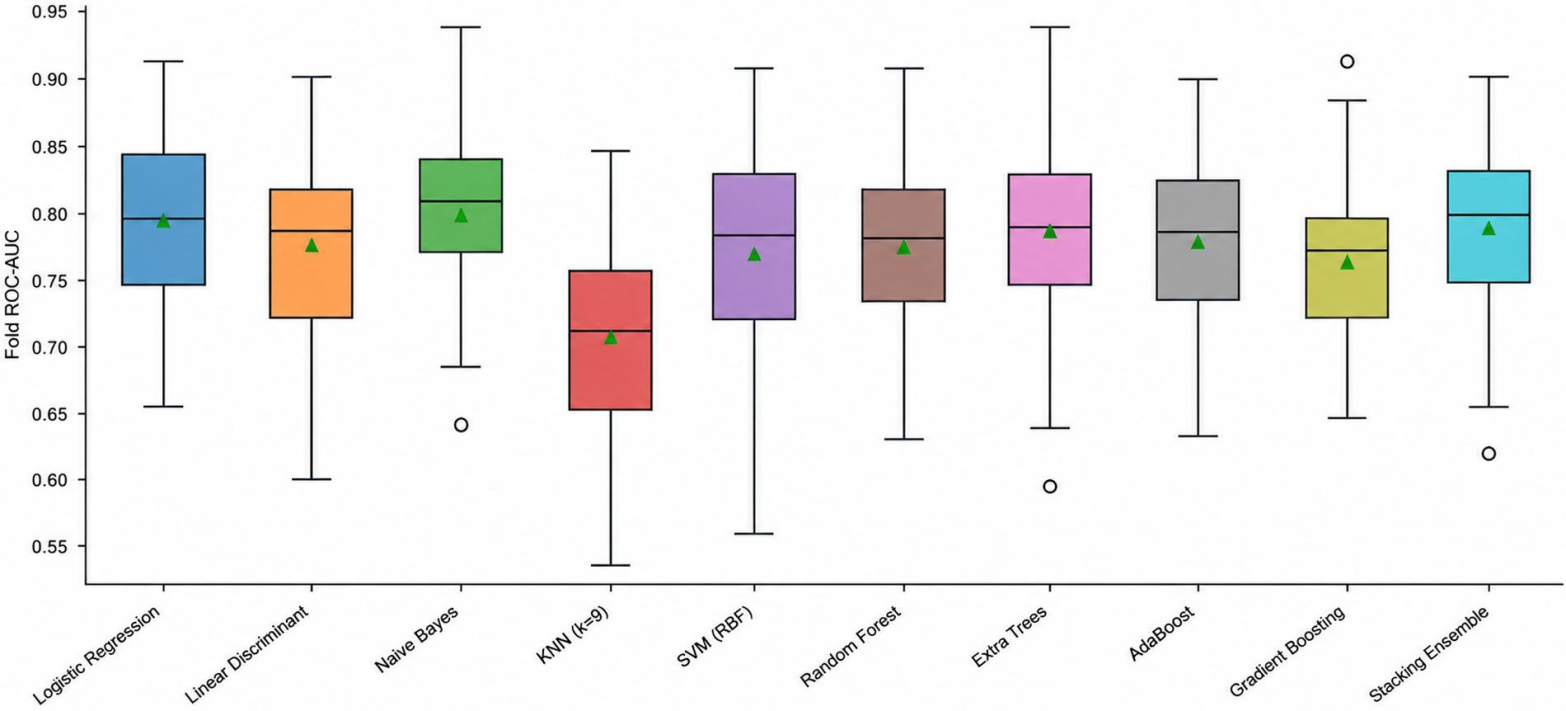
Cross-Validation stability comparison of Ten machine learning models.

Figure 10 presents the distribution of ROC-AUC values obtained from repeated cross-validation for all ten machine learning models. Overall, most algorithms demonstrated relatively stable predictive performance, with median ROC-AUC values ranging from approximately 0.71 to 0.81. Naive Bayes achieved the highest mean ROC-AUC (0.798 ± 0.061), closely followed by Logistic Regression (0.794 ± 0.061), Stacking Ensemble (0.789 ± 0.064), Extra Trees (0.787 ± 0.064), and AdaBoost (0.779 ± 0.063). These top-performing models exhibited overlapping ROC-AUC distributions, suggesting comparable discrimination performance. KNN demonstrated the lowest predictive performance and the widest relative spread, indicating reduced robustness across train–validation partitions. In contrast, AdaBoost, Logistic Regression, Random Forest, and Naive Bayes showed relatively compact distributions with limited variability, supporting the stability of their predictive performance. The absence of extreme performance fluctuations across repeated cross-validation evaluations suggests that the reported model performance was not driven by a particular data split and remained reasonably reproducible.

**Figure 11:**
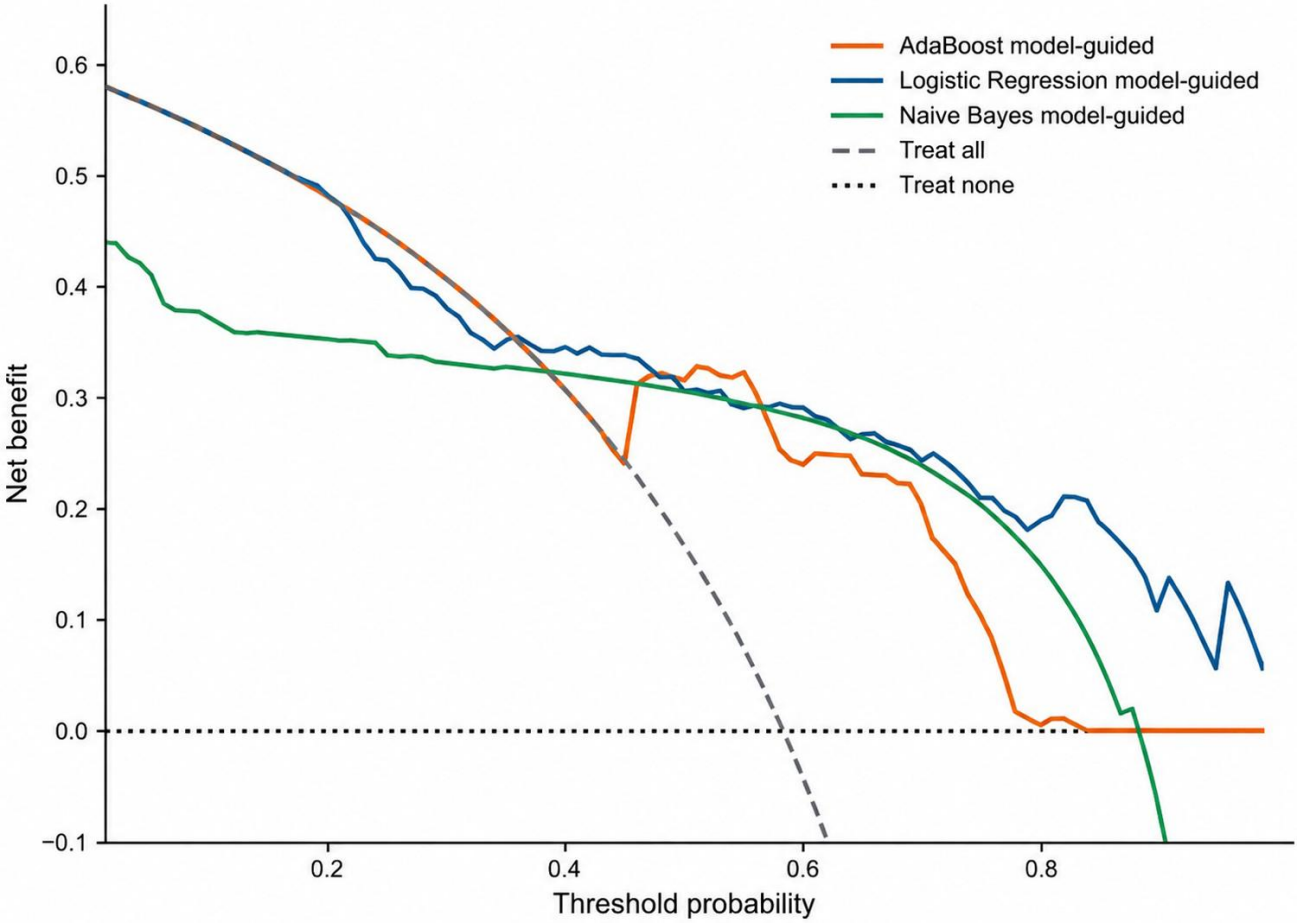
Decision curve analysis.

Decision curve analysis demonstrated positive net clinical benefit for all evaluated models across a range of threshold probabilities (Figure 11). However, Logistic Regression consistently achieved the highest net benefit across most clinically relevant thresholds (approximately 0.25–0.90), outperforming both the treat-all and treat-none strategies. AdaBoost provided moderate clinical utility but exhibited declining net benefit at higher thresholds, whereas Naive Bayes demonstrated favorable performance primarily in lower-threshold screening scenarios. These findings suggest that model discrimination alone may not fully capture clinical usefulness and highlight the importance of evaluating decision utility when selecting models for clinical deployment.

### 4.3 Explainability robustness across machine learning models

**Figure 12:**
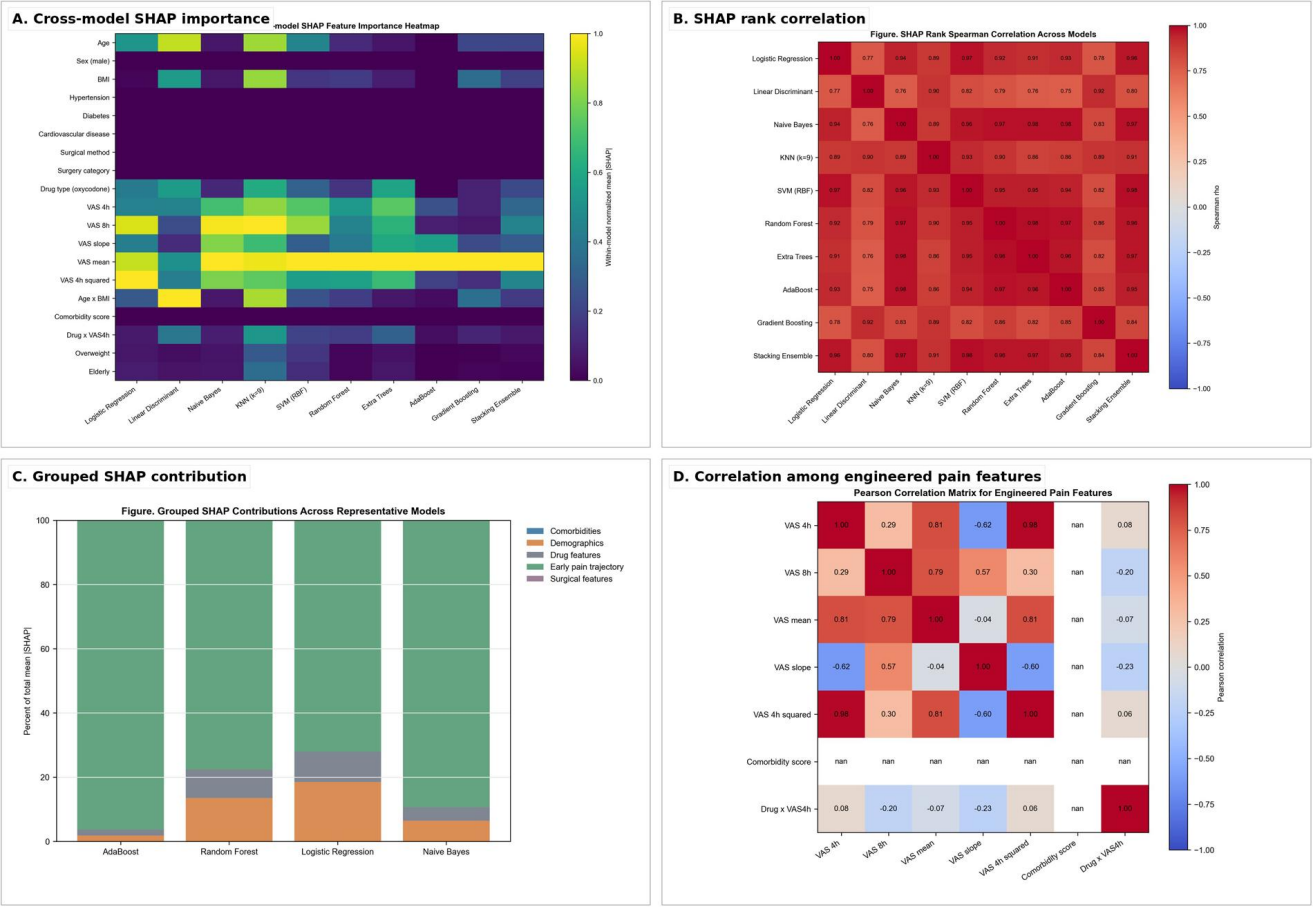
Explainability robustness across machine learning models. **(A)** Cross-model SHAP feature-importance heatmap showing normalized mean absolute SHAP values across the ten evaluated machine learning algorithms. **(B)** Pairwise Spearman rank correlation matrix of SHAP feature rankings across models, demonstrating the stability of feature attribution. **(C)** Grouped SHAP contribution analysis illustrating the relative importance of pain trajectory, demographic, comorbidity, surgical, and medication-related feature groups. **(D)** Pearson correlation matrix of engineered pain-related variables used to assess potential multicollinearity. Collectively, these analyses demonstrate that early postoperative pain trajectory features are consistently identified as the dominant predictors of poor analgesia across diverse machine learning approaches.

A comprehensive explainability analysis (Figure 12) was conducted across all ten machine learning algorithms to evaluate the robustness and consistency of feature attribution. Despite differences in model architecture and predictive mechanisms, a remarkably stable pattern of feature importance emerged.

Across virtually all models, early postoperative pain trajectory variables were consistently identified as the dominant predictors of poor analgesia. In particular, VAS mean, VAS measured at 4 h, VAS measured at 8 h, and VAS slope exhibited the highest SHAP contributions, indicating that both the magnitude and temporal evolution of early postoperative pain play a central role in determining subsequent analgesic outcomes. Among these variables, VAS mean demonstrated the greatest overall contribution and was consistently ranked among the most influential predictors across models.

To assess the stability of model explanations, SHAP feature rankings were compared across all evaluated algorithms. Pairwise Spearman rank correlations ranged from 0.75 to 0.98, demonstrating substantial agreement in feature importance ordering despite differences in predictive performance and model complexity. This finding suggests that the identified predictors represent robust clinical signals rather than artifacts of a specific modeling approach.

Grouped SHAP analyses further demonstrated that pain trajectory features accounted for the majority of explainable model information across representative algorithms. Depending on the model, variables describing early postoperative pain dynamics contributed approximately 70%–95% of the total SHAP importance, whereas demographic characteristics, comorbidities, surgical variables, and analgesic selection collectively explained a substantially smaller proportion of model predictions. These results reinforce the central clinical importance of early pain evolution in postoperative analgesia management.

Because several engineered variables were derived from the same underlying VAS measurements, additional correlation analyses were performed to evaluate potential multicollinearity. As expected, moderate-to-high correlations were observed among VAS-derived variables, particularly between VAS mean and its component measures. However, VAS slope exhibited substantially weaker correlations with VAS mean and captured complementary temporal information reflecting pain progression rather than static pain burden. Furthermore, the high consistency of SHAP rankings across ten independent models suggests that the overall interpretability conclusions remain robust despite the presence of correlated predictors.

Collectively, these findings demonstrate that the principal explanatory signals identified by the proposed framework are reproducible across multiple machine learning paradigms. The convergence of explainability results across models provides additional confidence that early postoperative pain trajectories represent genuine clinical determinants of analgesic failure and are not merely model-specific artifacts.

### 4.4 Comparative analysis: oxycodone vs. tilidine

**Figure 13:**
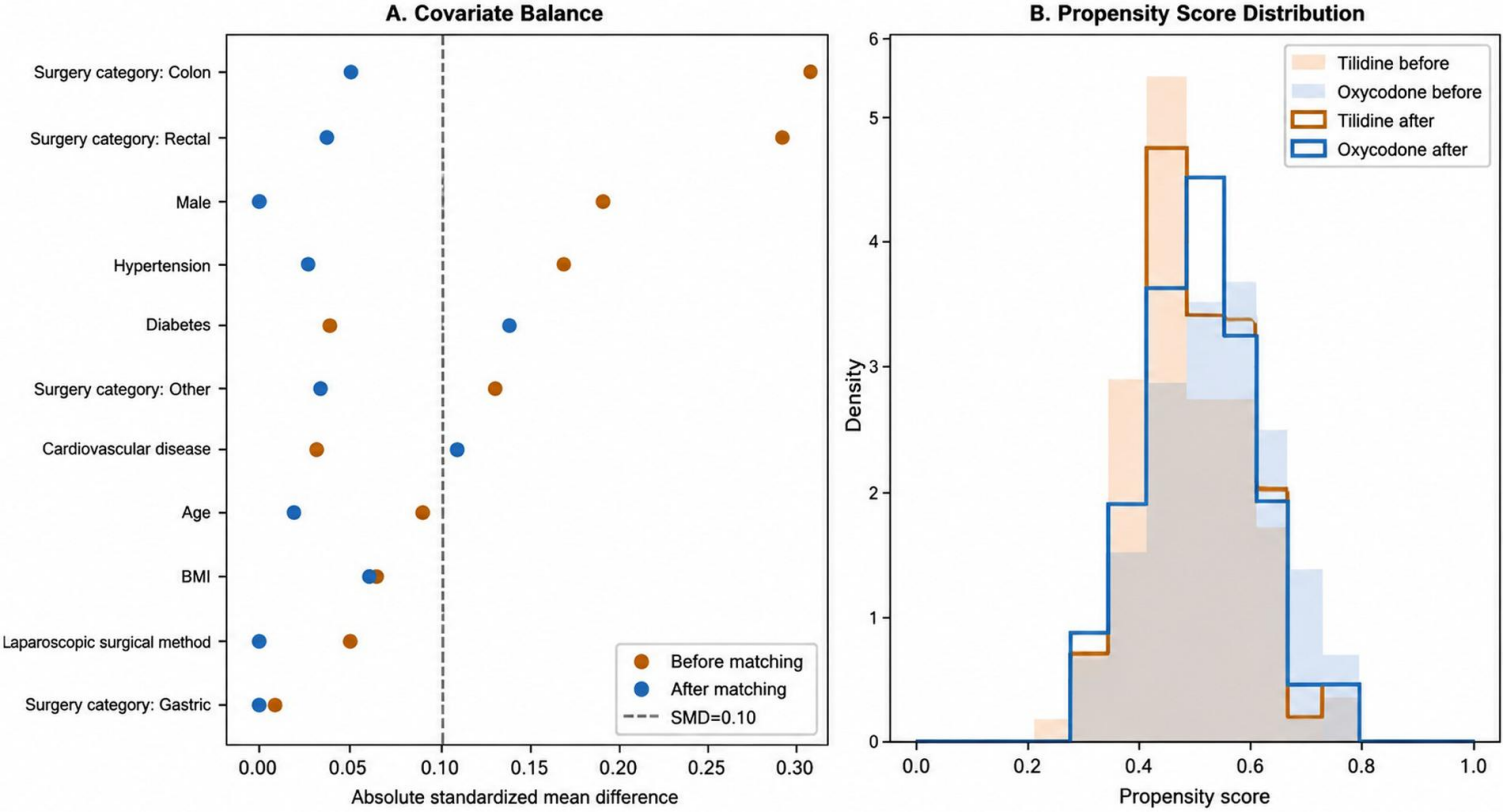
Propensity score matching diagnostics. (A) Covariate balance assessment before and after propensity score matching, showing the absolute standardized mean differences (SMDs) for baseline covariates. After matching, all covariates achieved improved balance with SMD values approaching or below the commonly accepted threshold of 0.10. (B) Propensity score distribution before and after matching between the tilidine and oxycodone groups, demonstrating improved overlap and comparability of patient characteristics after matching.

After matching, 79 patient pairs were retained. Covariate balance improved substantially, with the maximum absolute standardized mean difference decreasing from 0.308 before matching to 0.130 after matching. Despite improved balance, the difference in analgesic outcomes remained. The incidence of poor postoperative analgesia was 50.6% among patients receiving oxycodone compared with 67.1% among those receiving tilidine, corresponding to an absolute risk difference of 16.5 percentage points. Matched-pair analysis remained statistically significant (McNemar test, *p* = 0.041). These findings suggest that the observed association between analgesic type and postoperative analgesia outcome persisted after adjustment for measured baseline confounders. Covariate balance was further visualized using a Love plot and propensity score distribution plot (Figure 13) Figure 13A Love plot showing absolute standardized mean differences before and after 1:1 nearest-neighbor propensity score matching. Figure 13B Propensity score distributions of the oxycodone and tilidine groups before and after matching.

After matching, covariate balance improved substantially, with the maximum absolute standardized mean difference decreasing from 0.308 to 0.130.The Love plot showed that most covariates achieved improved balance after matching, and the propensity score distributions between the oxycodone and tilidine groups became more comparable after matching.

Comparative analysis demonstrated that patients treated with tilidine had a significantly higher risk of poor postoperative analgesia than those receiving oxycodone (68.3% vs. 48.5%). This difference was consistently reflected across both descriptive statistics and model-based predictions. Mean VAS scores were higher in the tilidine group at all postoperative time points (4h: 3.16 vs. 2.65; 8h: 2.79 vs. 2.23; 24 h activity: 3.02 vs. 2.70), indicating a persistently higher pain burden.

**Figure 14:**
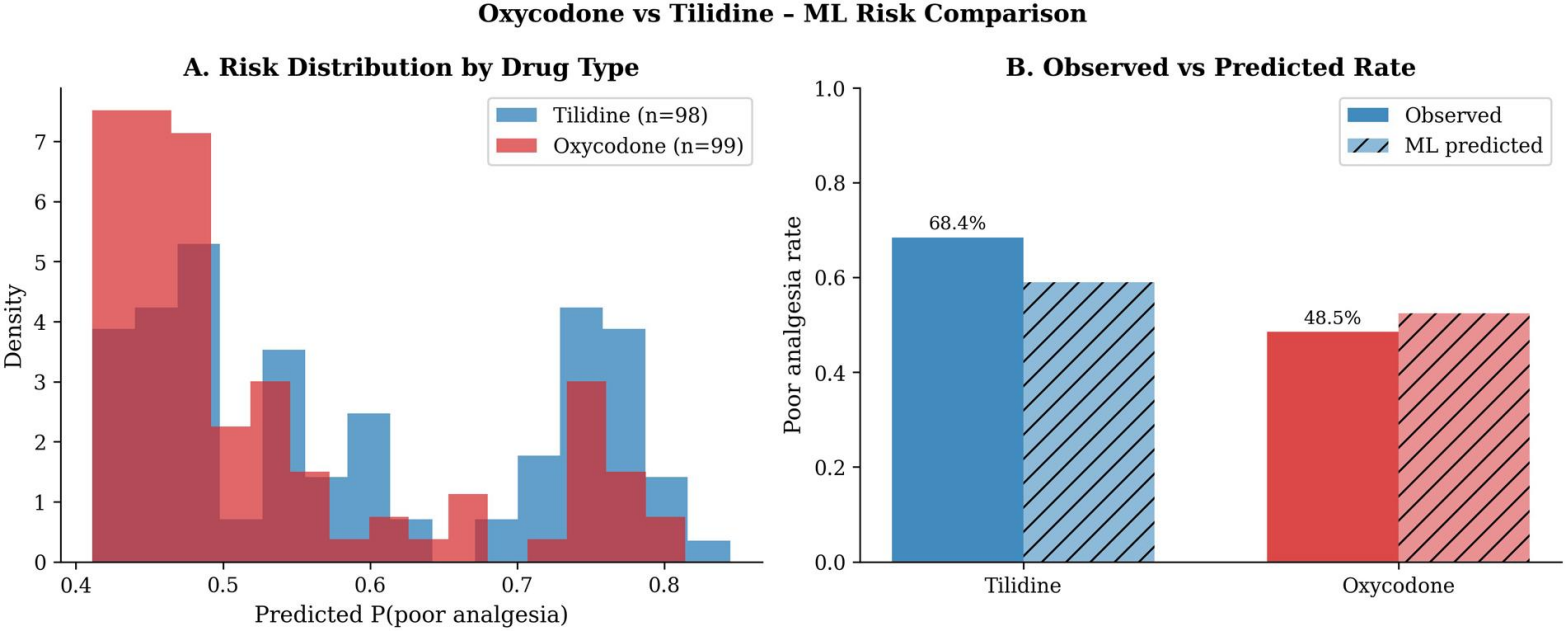
Oxycodone versus tilidine: machine learning–predicted risk comparison and observed analgesic outcomes. (A) Distribution of predicted probability of poor analgesia stratified by drug type, showing the model-estimated risk profiles for patients receiving tilidine and oxycodone. (B) Comparison of observed poor analgesia rates and corresponding predicted mean risks between the tilidine and oxycodone groups. The results demonstrate higher predicted risk and a higher observed rate of poor analgesic response in the tilidine group compared with the oxycodone group.

Importantly, the machine learning model identified drug type as an independent predictor of outcome after adjusting for demographic variables, comorbidities, surgical category, and early pain scores. To further evaluate whether the observed differences between oxycodone and tilidine could be explained by baseline patient characteristics, a propensity score matching analysis was performed. Propensity scores were estimated using age, sex, BMI, hypertension, diabetes mellitus, cardiovascular disease, surgery category, and surgical method. One-to-one nearest-neighbor matching was conducted using a caliper width of 0.2 standard deviations of the logit propensity score. Figure 14. illustrates both predicted risk distributions and observed outcome rates, showing consistent alignment between model predictions and empirical findings.

### 4.5 Non-linear effects and partial dependence

Partial dependence analysis revealed clear non-linear relationships between key continuous predictors and the probability of poor analgesia. For VAS at 4 h, the risk of poor analgesia increased sharply above a threshold of approximately 4, with a plateau effect observed at higher values. A similar but steeper relationship was observed for VAS at 8 h, confirming its role as a near-real-time risk indicator. BMI demonstrated a threshold effect above approximately 27 kg/m^2^, suggesting that overweight patients may be at increased risk, potentially due to altered pharmacokinetics or inflammatory profiles. Age showed a monotonic positive association with risk, although uncertainty increased in patients older than 75 years due to limited sample size.

**Figure 15:**
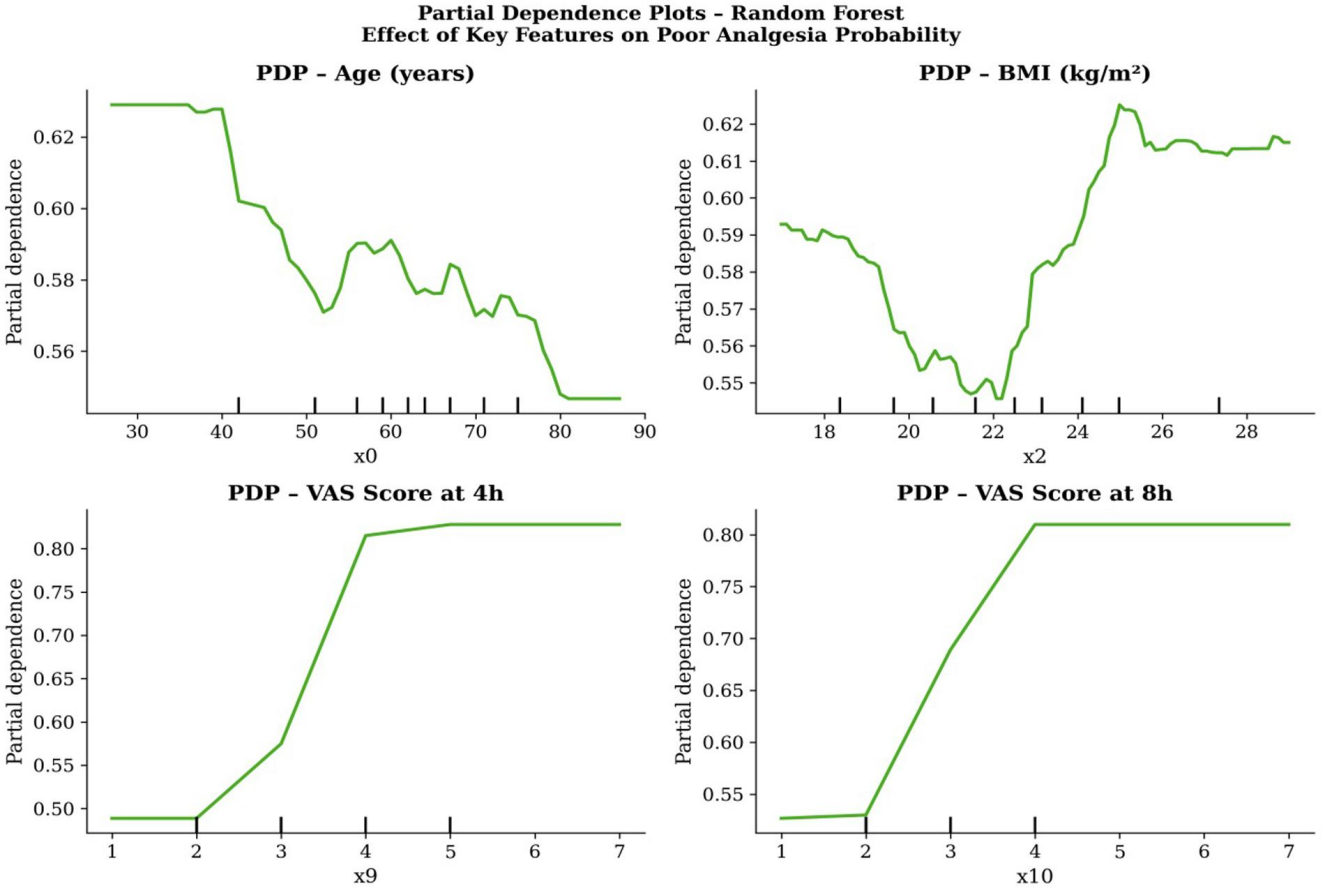
Partial dependence plots for age, BMI, VAS 4 h, and VAS 8 h. Non-linear threshold effects visible above VAS 4 h ≥ 4 and BMI ≥ 27.

To explore non-linear relationships between key predictors and the risk of poor analgesia, partial dependence plots were generated for selected variables (Figure 15). As illustrated, VAS scores at 4 h and 8 h exhibit clear threshold effects, with a marked increase in predicted risk when VAS values exceed approximately 4. This finding suggests that early postoperative pain intensity serves as a critical tipping point for subsequent analgesic failure. Similarly, BMI demonstrates a non-linear relationship, with increased risk observed above approximately 27 kg/m^2^, potentially reflecting altered pharmacokinetics or inflammatory responses in overweight patients. Age shows a gradual increase in risk, although with wider uncertainty at advanced ages. These results highlight the importance of capturing non-linear effects in predictive modeling and further support the use of machine learning approaches over traditional linear models.

These findings highlight the importance of non-linear modeling approaches and support the use of machine learning over traditional linear methods for capturing clinically relevant thresholds.

### 4.6 Case-based interpretability analysis

To further demonstrate the clinical utility and interpretability of the proposed ML–SHAP–LLM pipeline, four representative patient cases were selected from the cohort. These cases illustrate diverse postoperative pain trajectories, model predictions, and corresponding SHAP-based feature attributions. For each case (Table 4), model-derived risk estimates were combined with feature contribution analysis and LLM-generated clinical interpretation, enabling patient-level explanation of prediction outcomes.

**Table 4:** Representative cases with model predictions and SHAP-based interpretations.

Case	Clinical Profile	Drug	VAS 4 h/8 h (Slope)	Predicted Risk	Outcome	Key SHAP Interpretation
Case 1	61F, BMI 29.1, HTN + DM, colon resection	Tilidine	2/5 (+3)	77.30%	Poor	Rapid pain escalation (VAS slope +3) is the dominant driver
Case 2	59M, BMI 23.7, HTN, colon resection	Tilidine	5/2 (−3)	74.90%	Poor	High early pain burden (VAS mean 3.5) despite improvement
Case 3	72M, BMI 18.6, HTN, other abdominal	Tilidine	3/3 (0)	55.80%	Poor	Persistent moderate pain + vulnerability (age, low BMI)
Case 4	63M, BMI 23.6, DM, colon resection	Tilidine	3/2 (−1)	52.80%	Good	Moderate risk with improving pain trajectory

In Case 1, the patient exhibited a rapidly increasing pain trajectory between 4 and 8 h postoperatively (VAS slope +3), which was identified by the model as the strongest predictor of poor analgesia. SHAP attribution confirmed that the dynamic increase in pain intensity outweighed baseline factors such as comorbidities. This case highlights the importance of early pain escalation as a critical warning signal for subsequent analgesic failure.

In Case 2, despite a decreasing pain trajectory (slope −3), the model predicted a high risk of poor outcome. SHAP analysis revealed that the elevated early pain burden (VAS mean 3.5) remained the dominant factor, indicating that transient improvement does not necessarily mitigate risk when initial pain levels are high. In addition, the surgical category (colonic resection) contributed to increased predicted risk, suggesting procedure-specific vulnerability.

In Case 3, the patient demonstrated a stable but persistently moderate pain profile (VAS 3/3, slope 0). The model assigned intermediate risk, which was further influenced by patient-specific factors such as advanced age and low BMI. SHAP interpretation indicated that the absence of pain improvement, combined with increased physiological vulnerability, contributed to poor analgesic outcome. This case illustrates how non-dynamic pain patterns can still indicate risk when combined with demographic factors.

In Case 4, the model predicted moderate risk; however, the patient ultimately achieved a favorable outcome. SHAP attribution showed that although comorbidity (diabetes) contributed positively to risk, the decreasing pain trajectory (slope −1) and relatively low mean VAS mitigated the overall prediction. This case represents a borderline scenario and demonstrates that the model can capture nuanced interactions between improving pain patterns and baseline risk factors.

Across these cases, early postoperative pain trajectory emerged as the most influential determinant of outcome, with dynamic changes in VAS providing stronger predictive value than static measurements. Importantly, SHAP-based explanations enabled transparent identification of patient-specific risk drivers, while LLM-generated narratives translated these quantitative insights into clinically interpretable reasoning. Together, this case-based analysis demonstrates the potential of the proposed framework to support individualized postoperative pain management by integrating prediction, explanation, and clinical interpretation.

### 4.7 Sensitivity analysis using an alternative outcome definition

**Figure 16:**
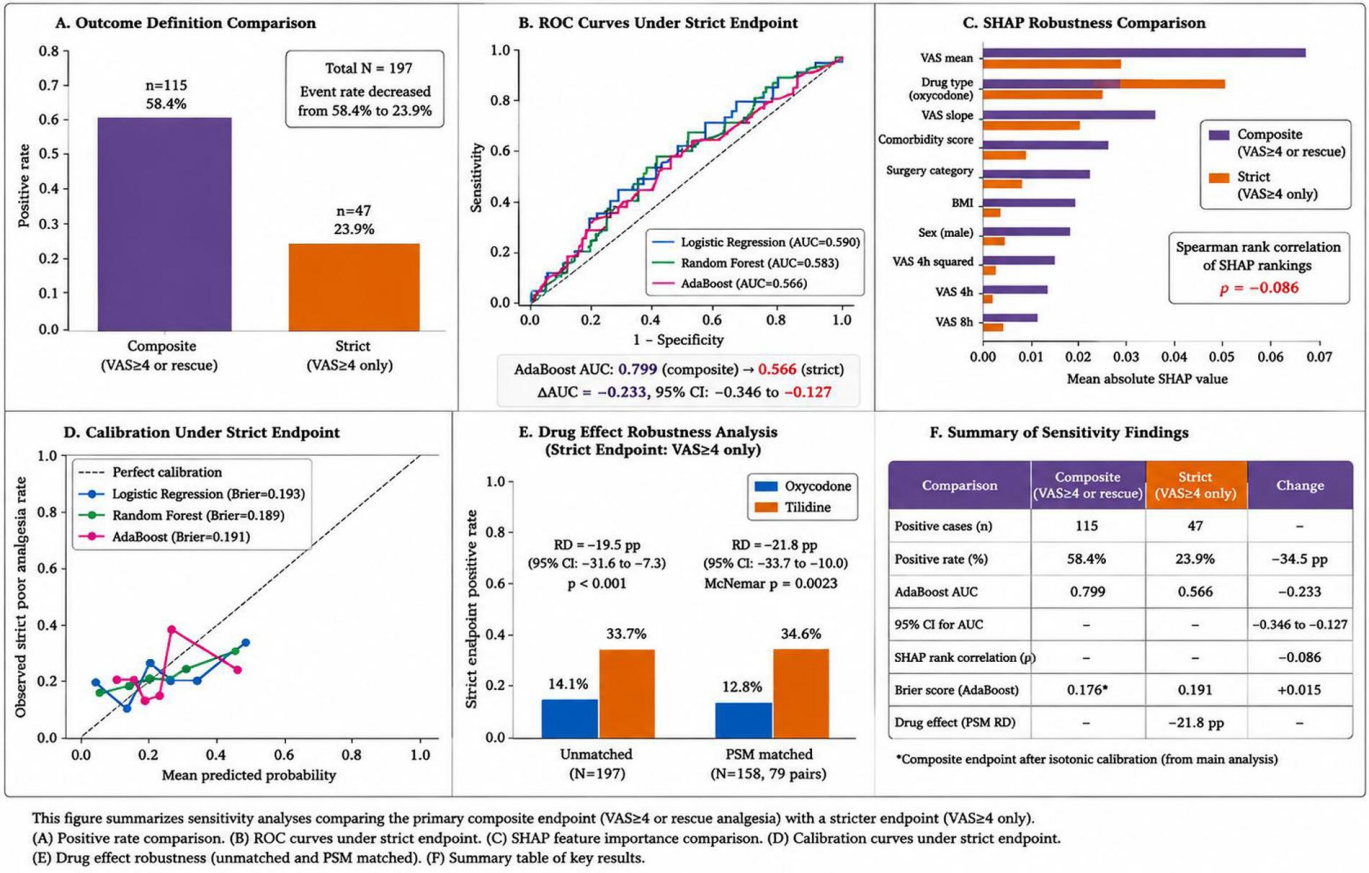
Sensitivity analyses evaluating the robustness of model performance under alternative outcome definitions and analytical approaches. (A) Comparison of outcome definitions between the composite endpoint (VAS ≥4 or rescue analgesia) and the strict endpoint (VAS ≥4 only), showing the corresponding positive case rates and sample distributions. (B) Receiver operating characteristic (ROC) curves under the strict endpoint definition for Logistic Regression, Random Forest, and AdaBoost models, with corresponding AUC values. (C) SHAP robustness comparison demonstrating the consistency of feature importance rankings between composite and strict endpoint definitions, with Spearman correlation analysis of SHAP rankings. (D) Calibration performance of machine learning models under the strict endpoint, comparing predicted probabilities with observed poor analgesia rates. (E) Drug effect robustness analysis using the strict endpoint, comparing poor analgesia rates between oxycodone and tilidine groups before and after propensity score matching. (F) Summary of sensitivity analysis findings, including changes in positive rate, model discrimination (AUC), SHAP ranking correlation, calibration performance, and estimated drug effects. Overall, the sensitivity analyses confirm the robustness of the main findings across alternative outcome definitions and analytical strategies.

To evaluate the impact of outcome definition on model performance, an additional sensitivity analysis (Figure 16) was performed using a stricter endpoint defined solely as activity VAS ≥ 4 at 24 h, excluding rescue analgesia from the outcome definition.

Under this stricter definition, the event rate decreased substantially from 58.4% (115/197) under the primary composite endpoint to 23.9% (47/197). Model performance declined across all evaluated algorithms. The AUC of AdaBoost decreased from 0.799 to 0.566 (ΔAUC = −0.233, 95% bootstrap CI: −0.346 to −0.127), while Logistic Regression and Random Forest achieved AUCs of 0.590 and 0.583, respectively.

Feature attribution patterns also differed considerably between the two outcome definitions. Comparison of SHAP rankings yielded a Spearman correlation coefficient of −0.086, suggesting that the predictors associated with the composite endpoint differed substantially from those associated with the VAS-only endpoint.

Despite these differences in predictive performance, the association between analgesic type and outcome remained consistent. Under the VAS-only definition, poor analgesia occurred in 14.1% of patients receiving oxycodone and 33.7% of patients receiving tilidine. Following propensity score matching, the corresponding rates were 12.8% and 34.6%, respectively, with matched-pair analysis remaining statistically significant (McNemar test, *p* = 0.0023).

A comprehensive summary of the sensitivity analyses is provided in Supplementary Figure S1. Sensitivity analyses were performed using a stricter endpoint defined solely as activity VAS ≥ 4 at 24 h, excluding rescue analgesia from the primary composite outcome. In this figure, (A) Comparison of event rates between the primary composite endpoint (VAS ≥ 4 or rescue analgesia) and the strict VAS-only endpoint. (B) ROC curves of the evaluated machine learning models under the strict endpoint definition. (C) Comparison of SHAP feature importance rankings between the primary and strict endpoint definitions. (D) Calibration performance of the machine learning models under the strict endpoint definition. (E) Robustness analysis of the association between analgesic type and outcome before and after propensity score matching. (F) Summary of key findings from the sensitivity analyses, including changes in event rate, model performance, feature attribution patterns, calibration, and drug-effect estimates.The sensitivity analyses demonstrated that outcome definition substantially influenced event prevalence, model discrimination, and feature importance patterns, whereas the observed association between oxycodone and improved analgesic outcomes remained consistent.

These findings suggest that the primary composite endpoint captures a broader clinical construct that includes both patient-reported pain burden and clinically meaningful analgesic escalation, whereas the VAS-only endpoint reflects a narrower measure of pain intensity.

### 4.8 Multicollinearity assessment and reduced-feature sensitivity analysis

**Figure 17:**
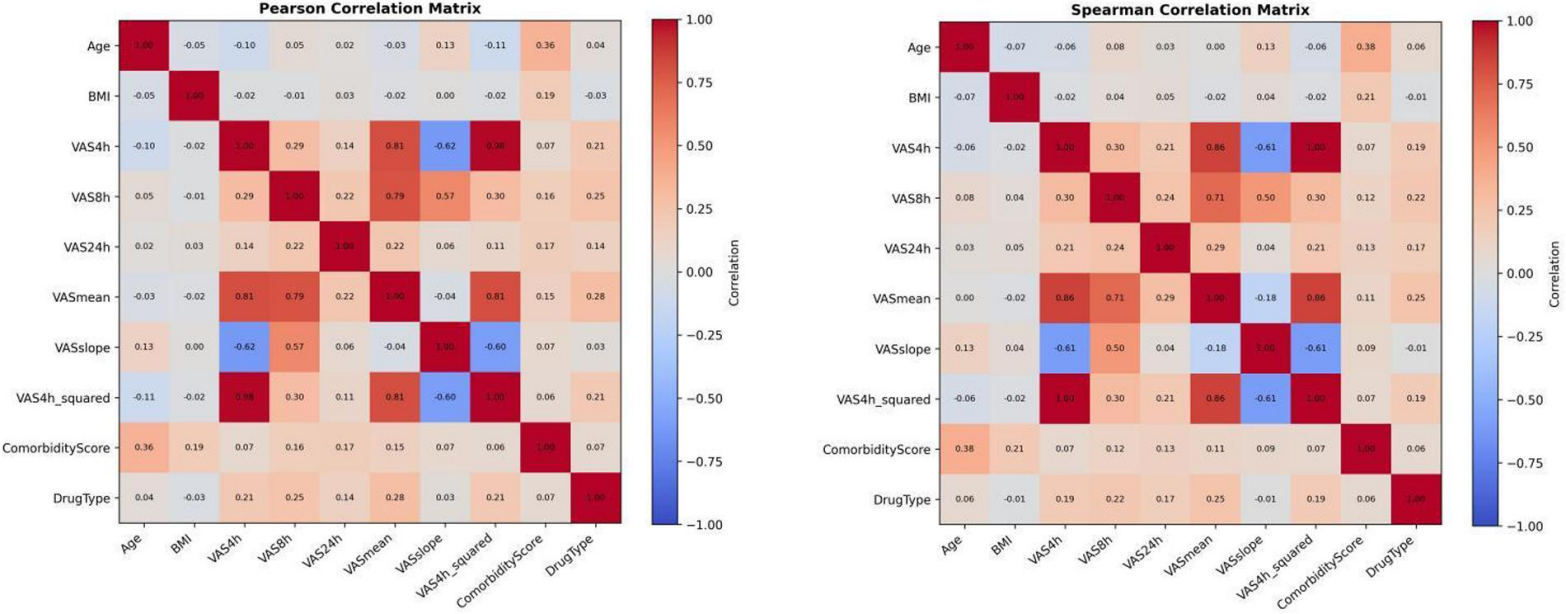
Pearson and spearman correlation analyses.

Pearson and Spearman correlation analyses were performed to evaluate potential multicollinearity among the candidate predictors (Figure 17). As expected, several engineered pain-related variables exhibited moderate-to-high correlations with their parent variables because they were mathematically derived from the same postoperative pain measurements. VASmean was strongly correlated with both VAS4 h (Pearson *r* = 0.81; Spearman *ρ* = 0.86) and VAS8 h (Pearson *r* = 0.79; Spearman *ρ* = 0.71), whereas VAS4h_squared demonstrated near-complete correlation with VAS4 h (Pearson *r* = 0.98; Spearman *ρ* = 1.00). VASslope showed moderate inverse correlation with VAS4 h (Pearson *r* = −0.62; Spearman *ρ* = −0.61).

To evaluate whether these correlations materially affected model performance and feature attribution, a reduced-feature sensitivity analysis was conducted after removing VASmean, VASslope, and VAS4h_squared. The AdaBoost model retained most of its predictive performance, with ROC-AUC decreasing from 0.790 to 0.755 and PR-AUC decreasing from 0.857 to 0.846. In addition, SHAP feature rankings remained largely preserved (Spearman correlation = 0.782), indicating that the dominant predictive signal was robust to the removal of correlated engineered variables.

These findings suggest that while multicollinearity exists among the VAS-derived predictors, the overall model performance and explainability conclusions are not driven by a single engineered feature and remain stable under feature reduction.

## 5 Discussion

In postoperative analgesia management, false-negative predictions (failure to identify patients who may experience inadequate analgesia) may result in untreated severe pain and delayed intervention, whereas false-positive predictions primarily lead to closer monitoring or rescue analgesic consideration. Therefore, a relatively lower decision threshold may be clinically acceptable. Based on this clinical context, we considered threshold probabilities between 20% and 60% as the clinically relevant range for postoperative analgesia risk stratification. Within this range, the AdaBoost model consistently demonstrated higher net benefit compared with the treat-all and treat-none strategies, supporting its potential clinical utility.

This study developed and comprehensively evaluated an explainable artificial intelligence framework for postoperative analgesia outcome prediction in patients undergoing abdominal surgery. Across ten machine learning algorithms, predictive performance was generally comparable, although important differences emerged when calibration, generalization, explainability robustness, and clinical utility were considered in addition to conventional discrimination metrics. Early postoperative pain trajectory variables consistently emerged as the dominant determinants of poor analgesia outcomes across models, highlighting the clinical importance of dynamic pain assessment during the early postoperative period. Furthermore, propensity score matching analyses supported a persistent association between analgesic type and postoperative pain outcomes, while decision curve analysis demonstrated that several models may provide meaningful clinical benefit across a range of clinically relevant decision thresholds. Collectively, these findings suggest that successful deployment of clinical prediction models requires a multidimensional evaluation framework extending beyond predictive accuracy alone.

### 5.1 Principal findings

In this study, we developed and evaluated an explainable machine-learning framework for predicting poor postoperative analgesia following abdominal surgery. Unlike many previous studies that focused primarily on discrimination performance, our framework systematically assessed discrimination, calibration, precision–recall performance, generalization ability, model stability, and explainability robustness across ten machine-learning algorithms.

Several important findings emerged. First, all leading machine-learning models demonstrated meaningful predictive value for identifying patients at risk of poor postoperative analgesia. Second, clinically informed feature engineering contributed substantially to model performance, particularly variables describing early postoperative pain trajectories. Third, explainability analyses consistently identified early pain-related features as the dominant determinants of subsequent analgesic outcomes across diverse modeling approaches. Finally, comparative analyses suggested that oxycodone was associated with a lower risk of poor analgesia than tilidine, an observation that remained robust across multiple sensitivity analyses.

Collectively, these findings support the feasibility of combining machine learning, explainable artificial intelligence (XAI), and large language model (LLM)-assisted communication to facilitate early postoperative pain risk stratification and personalized analgesic management.

### 5.2 Model performance, calibration, and clinical deployment

A major finding of this study is that model performance should not be evaluated solely on the basis of discrimination metrics such as ROC-AUC. Although several algorithms achieved similar predictive performance, important differences emerged when calibration, precision–recall performance, cross-validation stability, and learning behavior were considered simultaneously.

Among the evaluated algorithms, Naive Bayes achieved the highest average discrimination performance (AUC = 0.798 ± 0.061), followed closely by Logistic Regression (AUC = 0.794 ± 0.061) and Stacking Ensemble (AUC = 0.789 ± 0.064). However, discrimination alone did not fully characterize model quality. Logistic Regression demonstrated the lowest Brier score, indicating superior probability reliability, whereas Naive Bayes exhibited stronger discrimination but poorer calibration. Similarly, AdaBoost achieved competitive predictive performance while maintaining favorable specificity and explainability characteristics.

Precision–Recall analyses further demonstrated that all leading models substantially outperformed the no-skill baseline, indicating genuine positive-class discrimination rather than performance driven by outcome prevalence. Notably, Naive Bayes, Extra Trees, Logistic Regression, Random Forest, and AdaBoost achieved similarly high PR-AUC values, suggesting that multiple algorithms were capable of effectively identifying patients with poor postoperative analgesia.

Learning curve analyses provided additional insights into model robustness. Naive Bayes and Logistic Regression demonstrated the smallest train–validation performance gaps, indicating excellent generalization and minimal susceptibility to overfitting. AdaBoost exhibited moderate variance while maintaining stable validation performance. In contrast, Gradient Boosting, KNN, Random Forest, and Stacking Ensemble demonstrated larger train-validation gaps, suggesting a greater tendency toward overfitting despite favorable discrimination metrics. These findings highlight the importance of evaluating learning behavior when developing clinically deployable prediction models.

Cross-validation stability analyses revealed substantial overlap among the performance distributions of the leading algorithms. Although Naive Bayes achieved the highest mean validation AUC, the differences among Naive Bayes, Logistic Regression, AdaBoost, Extra Trees, Random Forest, and Stacking Ensemble were relatively modest. Consequently, no single algorithm overwhelmingly outperformed all others across every evaluation dimension. Instead, model selection should consider discrimination, calibration, stability, generalization, and interpretability simultaneously.

Interestingly, although AdaBoost was initially selected as the reference explainability model based on its strong discrimination performance in the primary analysis, subsequent calibration, learning-curve, and decision-curve analyses revealed that Logistic Regression demonstrated more favorable generalization and clinical utility characteristics. This observation highlights an important principle in clinical machine learning: the model with the highest discrimination performance is not necessarily the model that provides the greatest real-world clinical benefit. Therefore, model selection for clinical decision support should consider discrimination, calibration, stability, interpretability, and decision utility collectively rather than relying on a single performance metric.

Taken together, these results demonstrate that clinically useful machine-learning models should be evaluated using a multidimensional framework rather than relying exclusively on discrimination metrics.

### 5.3 Clinical significance of early pain trajectory

A particularly important finding was the dominant role of early postoperative pain dynamics in predicting subsequent analgesic outcomes. Across virtually all evaluated algorithms, VAS-related variables consistently emerged as the strongest predictors of poor analgesia.

Both pain intensity and pain evolution appeared clinically informative. Patients exhibiting persistently elevated pain scores or worsening pain trajectories during the early postoperative period were substantially more likely to experience inadequate analgesia at 24 h. In contrast, patients demonstrating rapid early pain improvement generally exhibited lower risk profiles.

These findings suggest that postoperative pain should be viewed as a dynamic process rather than a static measurement. Early pain trajectories may reflect multiple biological and clinical mechanisms, including inflammatory responses, individual pain sensitivity, surgical trauma burden, and differential response to analgesic treatment. Consequently, dynamic monitoring of pain evolution may provide more actionable information than isolated pain measurements obtained at a single time point.

From a clinical perspective, these results support the use of repeated early postoperative pain assessments to identify high-risk patients before clinically significant analgesic failure becomes established.

### 5.4 Explainability robustness across machine learning models

A major strength of the present study is the comprehensive evaluation of explainability robustness across ten machine-learning algorithms. Rather than relying on feature importance derived from a single model, we systematically compared SHAP-based explanations across diverse modeling approaches.

Despite substantial differences in algorithm architecture, a remarkably consistent pattern of feature importance emerged. VAS mean, VAS at 4 h, VAS at 8 h, and VAS slope were repeatedly identified as the dominant predictors of poor analgesia across virtually all models. Pairwise Spearman correlations of SHAP feature rankings ranged from 0.75 to 0.98, indicating strong agreement among models regarding the relative importance of predictors.

Grouped SHAP analyses further demonstrated that early pain trajectory variables accounted for the majority of explainable model information, whereas demographic characteristics, comorbidities, surgical factors, and medication-related variables contributed substantially less. This convergence across independent algorithms suggests that the identified predictors represent robust clinical signals rather than artifacts of a particular machine-learning method.

Because several engineered features were derived from the same underlying VAS measurements, additional correlation analyses were performed to assess potential multicollinearity. Although moderate correlations were observed among some pain-related variables, VAS slope demonstrated relatively independent behavior and captured complementary temporal information beyond static pain burden. Importantly, the consistency of SHAP rankings across models indicates that the overall interpretability conclusions remained robust despite the presence of correlated predictors.

Collectively, these findings provide strong evidence that early postoperative pain trajectories constitute genuine and reproducible determinants of analgesic outcomes.

### 5.5 Comparative effectiveness of oxycodone and tilidine

The comparative analysis revealed that patients receiving tilidine experienced a substantially higher rate of poor analgesia than those receiving oxycodone. This pattern was consistently observed across descriptive analyses and predictive modeling results, indicating a reproducible association between analgesic type and postoperative pain outcomes in the present cohort.

To further evaluate potential confounding by indication, an additional propensity score matching (PSM) analysis was performed. After balancing major measured baseline characteristics, including demographic variables, comorbidities, surgery category, and surgical method, the association between analgesic type and postoperative analgesia outcome persisted. In the matched cohort, the rate of poor analgesia remained higher among patients receiving tilidine than among those receiving oxycodone (67.1% vs. 50.6%), corresponding to a risk difference of 16.5 percentage points (McNemar test, *p* = 0.041).

The persistence of this association after propensity score matching suggests that the observed difference was not fully explained by the measured baseline characteristics available in the dataset. Nevertheless, because treatment allocation was not randomized, residual confounding from unmeasured factors, such as physician prescribing preferences, baseline pain sensitivity, perioperative analgesic management, and other clinical considerations, cannot be excluded. Therefore, the present findings should be interpreted as evidence of a robust observational association rather than a definitive causal effect. Future randomized controlled trials and advanced causal-inference studies will be required to determine whether the observed association reflects a true treatment effect.

### 5.6 Outcome definition and sensitivity analysis

To evaluate the robustness of the primary outcome definition, an additional sensitivity analysis was performed using a stricter endpoint defined solely as activity VAS ≥ 4 at 24 h, excluding rescue analgesia from the outcome definition.

Under this stricter definition, the event rate decreased substantially from 58.4% to 23.9%. Model performance also declined considerably, with the AUC of the AdaBoost model decreasing from 0.799 to 0.566 (ΔAUC = −0.233, 95% bootstrap CI: −0.346 to −0.127). Furthermore, feature attribution patterns differed markedly between the two endpoint definitions, with a Spearman correlation coefficient of −0.086 between SHAP rankings.

These findings suggest that the primary composite endpoint captures a broader clinical construct encompassing both patient-reported pain burden and clinically meaningful analgesic escalation. Rescue analgesia is frequently administered in response to inadequate pain control and therefore represents an important component of real-world postoperative pain management. The substantial decline in predictive performance under the VAS-only definition further indicates that early postoperative pain trajectories may be more predictive of subsequent analgesic intervention than of isolated pain scores measured at a single time point.

Importantly, the association between analgesic type and outcome remained consistent under the stricter endpoint definition. Patients receiving oxycodone continued to demonstrate lower rates of poor analgesia than those receiving tilidine, both before and after propensity score matching. Consequently, the primary composite endpoint was retained because it more closely reflects clinically actionable analgesic outcomes encountered in routine perioperative practice.

### 5.7 Value of explainable artificial intelligence

A major contribution of this study lies in the integration of explainable AI into the predictive framework. By applying SHAP-based feature attribution, we were able to identify not only the most important predictors at the population level but also patient-specific drivers of risk. This level of transparency is essential for clinical adoption, as it enables clinicians to understand and trust model outputs.

Interestingly, the baseline outcome-group comparisons demonstrated substantial differences in early postoperative pain variables and analgesic type, findings that were highly consistent with the SHAP-based explainability analyses. This concordance between conventional statistical comparisons and machine-learning feature attribution strengthens confidence in the robustness and clinical interpretability of the identified predictors.

Importantly, the explainability analysis revealed heterogeneity in risk drivers across patients. For example, some cases were dominated by dynamic pain escalation, while others were influenced by baseline vulnerability factors such as age and BMI. This heterogeneity underscores the limitations of one-size-fits-all analgesic strategies and supports the need for individualized pain management approaches.

### 5.8 Role of LLM in clinical interpretation

In addition to quantitative explainability, this study incorporated a large language model as an interpretation layer to translate structured model outputs into clinician-readable narratives. The LLM-generated explanations were consistent with SHAP-derived feature contributions and provided coherent summaries of patient-specific risk factors.

It is important to note that the LLM was not used for prediction but rather as a *post hoc* communication tool. This distinction ensures that the predictive validity of the system remains grounded in validated machine learning models while leveraging LLM capabilities to enhance interpretability and usability. By bridging the gap between numerical outputs and clinical reasoning, this approach facilitates integration into real-world workflows.

Although large language models have the potential to improve the interpretability and accessibility of machine learning outputs, they remain susceptible to hallucination, whereby generated statements may not be fully supported by the underlying input information. To mitigate this risk, the LLM in the present study was restricted to a *post hoc* explanatory role and received only structured model outputs, including predicted probabilities and SHAP-derived feature contributions. The model was explicitly instructed not to generate new diagnoses, treatment recommendations, causal inferences, or unsupported medical conclusions. In addition, all generated narratives underwent manual consistency verification against the original quantitative outputs to ensure that the reported explanations accurately reflected the predicted risk estimates and feature-attribution results.

Importantly, the LLM did not participate in model training, risk prediction, feature attribution, or clinical decision-making. Its function was limited to translating verified quantitative outputs into clinician-friendly natural-language explanations. Consequently, the LLM should be regarded as a communication and interpretation layer rather than a decision-support engine. This design substantially reduces the likelihood that hallucinated content could influence the predictive framework itself.

Nevertheless, manual verification does not completely eliminate the possibility of factual inconsistencies, and the reliability of LLM-assisted explanations remains an active area of investigation. Future studies should explore automated factuality-verification pipelines, retrieval-grounded explanation frameworks, and prospective clinician-centered evaluation protocols to further enhance the safety, transparency, and trustworthiness of LLM-assisted clinical interpretation systems.

### 5.9 Implications for clinical decision support

The proposed ML–SHAP–LLM pipeline represents a step toward clinically actionable decision support for postoperative pain management. By combining early risk prediction with transparent explanation and narrative interpretation, the system enables clinicians to identify high-risk patients within the first postoperative hours and adjust analgesic strategies accordingly.

Potential applications include early escalation of analgesic therapy, closer monitoring of high-risk patients, and optimization of drug selection based on individual risk profiles. The modular design of the system further supports its integration into electronic health records or perioperative monitoring platforms, enabling real-time clinical use.

### 5.10 Limitations

Several limitations should be acknowledged when interpreting the findings of this study.

First, this was a retrospective single-center study with a relatively modest sample size. Although extensive internal validation procedures were performed, including repeated cross-validation, calibration assessment, learning curve analysis, sensitivity analyses, and explainability robustness evaluations, the possibility of model optimism and limited generalizability cannot be completely excluded. External validation using independent cohorts from other institutions will be necessary to confirm the reproducibility and transportability of the proposed framework. An additional limitation is that the pain slope variable may be influenced by rescue analgesic interventions occurring between the 4-hour and 8-hour assessments. Because detailed time-stamped rescue analgesia records were not available, the observed pain trajectory reflects the combined effects of pain progression and therapeutic intervention. Consequently, $\mathrm{VA}S_{\mathrm{slope}}$ should be interpreted as a real-world indicator of postoperative pain evolution rather than a purely physiological measure of untreated pain dynamics. Future prospective studies incorporating high-resolution medication timestamps may enable dynamic pain trajectory modeling.

Second, the observational design limits causal interpretation. Although propensity score matching was performed to reduce measured baseline imbalances when comparing oxycodone and tilidine, residual confounding arising from unmeasured clinical factors, physician prescribing preferences, perioperative management differences, or patient-specific characteristics may still be present. Therefore, the observed associations between analgesic type and postoperative outcomes should be interpreted as robust observational findings rather than definitive causal effects.

Third, the study focused primarily on structured clinical variables and early postoperative pain measurements. Other potentially relevant predictors, including psychological factors, preoperative pain sensitivity, socioeconomic characteristics, genetic variability, inflammatory biomarkers, and intraoperative physiological signals, were not available in the current dataset. Incorporation of multimodal perioperative data may further improve predictive performance and clinical applicability.

Fourth, several engineered variables were derived from the same underlying pain assessments, introducing the possibility of feature correlation and multicollinearity. Although additional correlation analyses, grouped SHAP analyses, and cross-model explainability evaluations demonstrated consistent feature attribution patterns across diverse machine learning algorithms, the interpretation of individual feature contributions should nevertheless be approached with caution. Future studies should investigate causal feature attribution methods and longitudinal modeling approaches to further disentangle correlated predictors.

Fifth, while the explainability analyses demonstrated high consistency across multiple machine learning models, explainability methods inherently provide approximations of model behavior rather than direct evidence of biological causation. Consequently, the identified predictors should be interpreted as clinically informative risk markers rather than mechanistic determinants of analgesic outcomes.

Finally, the large language model component was incorporated solely as a *post hoc* communication interface and was not involved in risk prediction. Although structured prompts, restricted inputs, and manual verification procedures were used to minimize hallucination risk, LLM-generated narratives remain susceptible to factual inaccuracies. Future work should incorporate automated factuality verification, prospective clinician evaluation, and human–AI interaction studies to ensure safe deployment in real-world clinical environments.

Despite these limitations, the study provides a comprehensive evaluation of predictive performance, calibration, stability, generalization, and explainability across multiple machine learning paradigms, offering a transparent and clinically interpretable framework for early postoperative analgesia risk stratification.

### 5.11 Future directions

Several promising directions may further enhance the clinical utility and scientific value of the proposed framework.

First, future studies should prioritize prospective multicenter validation to evaluate model transportability across diverse patient populations, surgical procedures, and healthcare settings. Although the present study demonstrated encouraging internal robustness through repeated cross-validation, calibration analysis, learning curve assessment, and explainability consistency evaluations, external validation remains essential before routine clinical deployment.

Second, the current framework could be expanded through the integration of multimodal perioperative data. In addition to structured clinical variables and pain assessments, future models may incorporate intraoperative physiological signals, laboratory biomarkers, wearable monitoring data, medication administration records, and patient-reported outcomes. Such multimodal approaches may improve predictive accuracy while providing a more comprehensive representation of postoperative recovery trajectories.

Third, the findings highlight the importance of pain dynamics rather than isolated pain measurements. Future work should therefore investigate longitudinal and time-series modeling approaches, including recurrent neural networks, temporal transformers, and continuous monitoring frameworks, to better characterize the evolution of postoperative pain and enable real-time risk prediction throughout the perioperative period.

Fourth, although SHAP-based explainability demonstrated strong consistency across multiple machine learning algorithms, future research should explore causal inference and counterfactual explainability approaches. These methods may provide deeper insights into whether identified predictors merely correlate with poor analgesia or actively contribute to its development, thereby improving the clinical actionability of model explanations.

Fifth, the observed association between oxycodone and improved analgesic outcomes warrants further investigation using randomized controlled trials and advanced causal inference methodologies. Such studies may help determine whether analgesic selection itself influences postoperative pain trajectories or whether the observed associations are partially attributable to residual confounding.

Sixth, the LLM-assisted interpretation layer may be further developed into an interactive clinical decision-support interface. Future systems could provide personalized risk explanations, evidence-based recommendations, uncertainty estimation, and clinician feedback mechanisms while incorporating automated factuality verification to minimize hallucination risk and ensure safe deployment.

Finally, future research should move beyond prediction toward intervention. By combining early risk detection, explainable machine learning, and adaptive clinical decision support, it may become possible to establish closed-loop perioperative pain management systems capable of dynamically adjusting analgesic strategies according to individual patient risk profiles. Such systems have the potential to advance precision perioperative medicine and improve postoperative recovery outcomes.

## 6 Conclusion

This study presents a comprehensive explainable artificial intelligence framework for postoperative analgesia outcome prediction and clinical decision support in abdominal surgery. Across ten machine learning algorithms, early postoperative pain trajectories emerged as the most influential determinants of analgesic outcomes, underscoring the importance of dynamic pain assessment during the immediate postoperative period. Beyond conventional discrimination metrics, additional analyses incorporating calibration assessment, precision–recall evaluation, learning-curve analysis, cross-validation stability testing, explainability robustness assessment, sensitivity analyses, and decision curve analysis demonstrated that model reliability and clinical utility should be considered alongside predictive performance when selecting models for clinical deployment.

Propensity score matching analyses further supported a robust observational association between analgesic type and postoperative pain outcomes, although causal inference cannot be established in this retrospective study design. By integrating machine learning, explainable artificial intelligence, and large language model–assisted interpretation, the proposed framework provides a transparent and clinically actionable approach for postoperative pain risk stratification and individualized perioperative decision support. Future multicenter prospective studies and external validation cohorts are warranted to confirm generalizability, evaluate clinical impact, and facilitate real-world implementation.

## Data Availability

The dataset used in this study contains patient-level clinical information and is subject to ethical and privacy restrictions. Due to institutional regulations and data protection policies, the data are not publicly available. Access to the dataset may be granted upon reasonable request to the corresponding author, subject to approval by the institutional ethics committee and compliance with data sharing agreements.. Requests to access the datasets should be directed to Junting Wu, wujunting222@outlook.com.
