# Supplementary material for: Explainable artificial intelligence for postoperative analgesia risk stratification and clinical decision support in abdominal surgery
Source: Front Digit Health. 2026 Aug 3;8:1851084. doi: 10.3389/fdgth.2026.1851084 (PMC13478206; doi:10.3389/fdgth.2026.1851084)
Supplement: Supplementary file 1 [file Table1.docx]

Appendix A: Key Implementation Code

A1. Feature Engineering

df['BMI'] = df['Weight'] / (df['Height']/100)**2

df['VAS_slope'] = df['VAS8h'] - df['VAS4h']

df['VAS_mean'] = (df['VAS4h'] + df['VAS8h']) / 2

df['Comorbidity_score'] = df['HTN'] + df['DM'] + df['CVD']

A2. Outcome Definition

df['PoorAnalgesia'] = (

(df['VAS24h_activity'] >= 4) |

(df['RescueCount'] >= 1)

).astype(int)

A3. Model Training (Example: AdaBoost)

from sklearn.ensemble import AdaBoostClassifier

from sklearn.model_selection import StratifiedKFold, cross_val_predict

from sklearn.metrics import roc_auc_score

model = AdaBoostClassifier(n_estimators=100, learning_rate=0.5)

cv = StratifiedKFold(n_splits=5, shuffle=True, random_state=42)

proba = cross_val_predict(model, X, y, cv=cv, method='predict_proba')[:,1]

auc = roc_auc_score(y, proba)
